# Contrasting patterns for bacteria and archaea in response to salt stress across alpine wetlands of the Tibetan Plateau

**DOI:** 10.1016/j.fmre.2024.02.010

**Published:** 2024-03-07

**Authors:** Xu Liu, Manuel Delgado-Baquerizo, Teng Yang, Gui-Feng Gao, Yu Shi, Haiyan Chu

**Affiliations:** a State Key Laboratory of Soil and Sustainable Agriculture, Institute of Soil Science, Chinese Academy of Sciences, Nanjing 210008, China; b University of Chinese Academy of Sciences, Beijing 100049, China; c Laboratorio de Biodiversidad y Funcionamiento Ecosistémico. Instituto de Recursos Naturales y Agrobiología de Sevilla (IRNAS), CSIC, Sevilla E-41012, Spain; d State Key Laboratory of Crop Stress Adaptation and Improvement, School of Life Sciences, Henan University, Kaifeng 475004, China

**Keywords:** Alpine wetlands, Stress-gradient hypothesis, Prokaryotes, Biodiversity, Ecological networks, Soil nature conservation

## Abstract

Understanding microbial responses to environmental stress is crucial for comprehending their distribution and supporting conservation efforts. Yet, comprehensive evaluations of these responses across diverse microbial taxa within the framework of classical ecological theories are scarce. This gap limits our ability to predict the impact of environmental changes on the diversity and functions of soil microbes in natural settings. In this study, we conducted a field survey across twenty alpine wetlands on the Tibetan Plateau. Employing amplicon sequencing with network theories, we investigated the biodiversity and coexistence of bacteria and archaea under a wide range of natural salinity conditions. Our results demonstrated a linear decrease in bacterial diversity with increased salinity, while archaeal diversity showed a non-linear pattern, initially declining and then rising, reflecting varied adaptation strategies to salt stress. Network analysis revealed a heightened complexity in positive associations among bacteria under salt stress. In contrast, archaea exhibited a decrease in both positive and negative associations, with the community succession to halophiles. These results imply that bacteria may counteract stress through enhanced facilitation, whereas archaea predominantly rely on stress-tolerant taxa. Additionally, structural equation modeling confirmed our hypothesis regarding the ecological response strategies of bacteria and archaea to salinity stress, showing that the variation in bacterial diversity is mainly explained by the complexity of positive associations, whereas archaeal diversity directly correlates with salinity levels. Overall, this study offers novel insights into the ecological strategies of prokaryotes under salinity stress and enriches our understanding of the processes maintaining microbial diversity in stressful conditions.

## Graphical abstract

**Figure:**
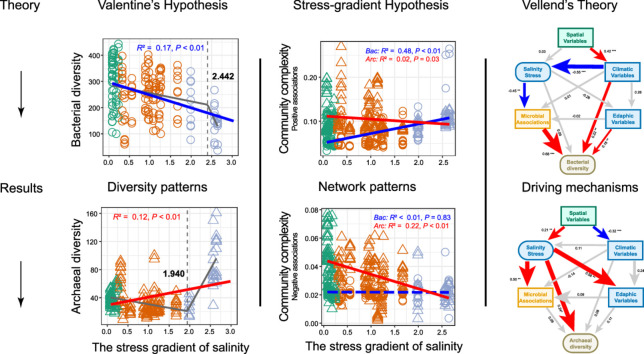

## 1 Introduction

Bacteria and archaea, as key prokaryotic groups in the Tree of Life, play crucial roles in ecosystem functioning, including biodiversity maintenance and nutrient cycling processes like ammonia oxidation [1,2]. Understanding how these microbes respond to environmental stressors is essential for comprehending their distribution and promoting conservation efforts [3], [4], [5]. Chronic energy stresses, such as salinity, significantly influence the ecology and evolution of bacteria and archaea [6], [7], [8]. The response to stress in these two groups is anticipated to vary due to their unique physiological metabolisms and distinct evolutionary histories [9]. Bacteria, for instance, typically focus on exploiting a variety of resources. In contrast, archaea are equipped with specialized adaptations, such as mechanisms to manage high sodium influx, enabling them to maintain cellular functionality and thrive in environments with elevated salt concentrations [10]. While a few studies have focused on the importance of regional salinity stress for individual microbial groups, there is a notable lack of empirical evidence examining the concurrent changes in biodiversity and coexistence of bacteria and archaea under a broad range of salinity stress, particularly in natural ecosystems. Such information would provide valuable information about the response of microbial taxa and their interactions, as well as the ecological strategies adopted by bacteria and archaea in coping with stress.

Despite progress in the understanding of microbial responses to salinity stress, several gaps in knowledge remain. First, many studies exploring the relationship between microbes and stress focus on the biochemical traits of a single group of organisms, such as the metabolic pathways of extremophiles [11,12]. Although these studies are important in understanding the selective force of stress at the strain level, they do not provide a comprehensive understanding of the ecological unity and difference between bacteria and archaea at the community level. Second, microbes exist in complex networks and perform functions through cooperation and competition among species in the networks [13]. For example, soil organisms require cooperative interactions among multiple species to support organic matter decomposition [14]. However, with some views suggesting that competition is more likely in soil environments, our understanding of the response of microbial interactions to stress is still limited [15]. Especially, recent researches have advanced the understanding of microbial ecological coexistence, leveraging network analysis to furnish compelling evidence for such associations [14,^16^,17]. Furthermore, the approach of discerning associations, grounded in community assembly theory, which encompasses environmental selection, dispersal limitation, and biotic filtering, yields insights into potential biotic interactions inferred from coexistence patterns [18]. This emerging viewpoint enriches the comprehension of how microbial interactions contribute to the maintenance of biodiversity and functional performance. Moreover, the classical stress gradient hypothesis in ecology, which mostly applies to plants and animals, suggests that competitive associations switch to positive associations with increasing stress [19]. Bacteria and archaea are far more complex than plants and animals, so their responses to stress may have more possibilities [20,21]. Third, many stress studies categorize stress into low and high levels, but the impact of the wide stress gradient on microbes is less well described [22,23]. In particular, microbes will demonstrate realistic response patterns along naturally occurring stress gradients compared to laboratory environments. Identifying the threshold between stress and microbes is critical for comprehending how microbes non-linearly respond to stress. Finally, most studies on soil organisms have mainly focused on environmental factors affecting diversity. However, the extent to which ecological networks shape microbial taxa, particularly under salinity stress, is poorly understood. This is important for updating the mechanisms of maintaining microbial diversity in harsh conditions.

Thus, we conducted a field survey across a distance of approximately 1,200 km in 20 alpine wetlands located on the Tibetan Plateau, with an average elevation of over 4,000 meters above sea level. Especially these alpine wetlands encompassed a wide range of salt stress levels, varying from 0.2 to 440 practical salinity units (psu), and could be categorized into low-stress (freshwater wetlands with salinity levels ≤ 1 psu), medium-stress (brackish wetlands with 1 < salinity ≤ 35 psu), and high-stress (saline wetlands with salinity > 35 psu) environments according to well-established wetland classifications [24,25]. This naturally occurring gradient of salt stress offers an ideal framework for investigating the adaptive responses of bacteria and archaea across a wide spectrum of environmental stressors. By utilizing the amplicon-based microbial communities and bioinformatics along the stress gradient, we aim to address the following questions:

(i) Does the diversity of bacteria and archaea follow different biogeographical patterns with increasing salinity stress? Based on previous reports about prokaryotic traits under persistent stress [9], we hypothesize that the diversity of bacteria and archaea respond differently to salt stress.

(ii) Do the coexistence patterns of bacteria and archaea differ across salinity stress gradients? Given that the general exception of the stress gradient hypothesis could be reconciled with microbial traits [26], we assume that bacteria and archaea may exhibit contrasting paradigms of network associations to survive salinity stress.

(iii) How does stress alter the relationship between microbial diversity and ecological networks? Despite biotic associations being largely neglected as contributors to biodiversity [27], we suppose that the importance of ecological networks may differ between bacteria and archaea under stress due to their unique life-history characteri-stics.

## 2 Materials and methods

### 2.1 Study area and field sampling

**Fig. 1:**
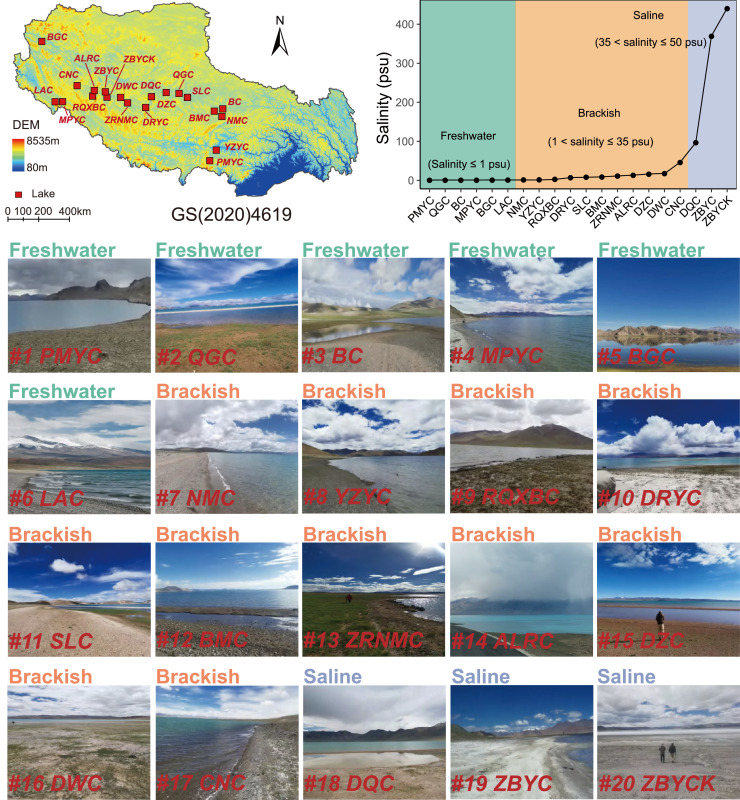
**Maps and photographs of involved fields in alpine wetlands of the Tibetan Plateau.** The alpine wetlands were located on the Tibetan Plateau in China, labeled as: #1: PMYC, #2: QGC, #3: BC, #4: MPYC, #5: BGC, #6: LAC, #7: NMC, #8: YZYC, #9: RQXBC, #10: DRYC, #11: SLC, #12: BMC, #13: ZRNMC, #14: ALRC, #15: DZC, #16: DWC, #17: CNC, #18: DQC, #19: ZBYCKN, #20: ZBYCKS, respectively. The wetlands have been classified into three levels of salinity stress: freshwater (salinity ≤ 1 psu), brackish (1 < salinity ≤ 35 psu), and saline (salinity > 35 psu), based on the definition of Liu et al. [24]. The subfigures are presented in a progressive order, starting from the lowest stress level (freshwater) and proceeding through medium stress (brackish) to high stress (saline).

We conducted a standardized field survey of 20 alpine wetlands located on the Tibetan Plateau (Fig. 1). These wetlands are characterized by high elevation location (above 4,224 m.a.s.l) and adjoined to lake shores, with the area being at least 50 km^2^. These wetlands were affiliated with Yamdrok Tso (YZYC), Puma Yum Co (PMYC), Mapam Yum Co (MPYC), Raksas Tal Co (LAC), Bangong Co (BGC), Ruldan Tso (CNC), Chovo Tso (RQXBC), Ang Laren Co (ALRC), North Tabya Tsaku (ZBYCKN), South Tabya Tsaku (ZBYCKS), Dawa Co (DWC), Zhari Nam Co (ZRNMC), Tangra Yum Co (DRYC), Dangqiong Co (DQC), Dagze Co (DZC), Jarg Co (QGC), Siling Co (SLC), Pam Tso (BMC), Bong Co (BC), and Nam Co (NMC), which covered the geographical range from 79.82° to 91.11°*E* longitudinally and from 28.63° to 33.45°*N* latitudinally over 1,200 km. These alpine wetlands consist of various types of alpine grasslands, such as *Kobresia humilis, K. pygmaea*, and *K. tibetica*. The dominant soil types, as classified by the Chinese Soil Genetic Classification System, include bog soils, peat soils, meadow soils, and frigid plateau solonchaks. These soils are characterized by a loamy texture, exhibiting an alkaline pH and superior water retention capabilities.

All sampling occurred during a short time interval from July 5^th^ to 20^th^, 2020, with no extreme weather occurring during the sampling period. At each wetland, 10 replicates of soil composite samples were collected along the lakeshore at a depth of 10 cm, with a minimum of 100 meters between samples to avoid local-scale spatial autocorrelation. At least 5 soil cores mixed as one composite sample. Litter and detritus were removed from the soil cores before they were placed in sterile plastic bags. This resulted in a total of 200 samples (20 wetlands × 10 replicates). The sample sites were recorded and photographed. Further details about the sample sites can be found in Table S1. The samples were divided into two parts, with one part being stored at -80 °C for molecular analysis and the other part being stored at 4 °C for physicochemical analysis.

### 2.2 Physicochemical analyses and data collection

In this study, nine edaphic variables were measured for all 200 soil samples collected from 20 alpine wetlands on the Tibetan Plateau. These variables included soil pH, dissolved organic carbon (DOC), dissolved organic nitrogen (DON), nitrate nitrogen (NO_3_^−^-N), ammonium nitrogen (NH_4_^+^-N), total carbon (TC), total nitrogen (TN), total potassium (TK), and total phosphorus (TP). The soil pH was determined in a 1:5 soil/water suspension using a Thermo Orion-868 pH meter (Thermo Orion-868, MA, USA). DOC was extracted by adding 50 mL of distilled water to 5 g of samples, shaking for 1 h, and vacuum filtering through a 0.45 µm PES filter (Fisher). Then, the concentration of DOC was determined using a total organic carbon analyzer (Multi N/C 3100, Analytik Jena AG, Germany). The content of NH_4_^+^-N, NO_3_^−^-N, and dissolved total nitrogen (DTN) were extracted using a ratio of 5 g samples to 50 mL 2 M KCl. After shaking for 1 hour, the extracts were filtered using a 0.45 µm PES filter, and the concentrations were measured using a continuous flow analyzer (San ++ System, Skalar, Holland). The DON was calculated using the following formula: DON = DTN − NH_4_^+^-N − NO_3_^−^-N). The contents of TC and TN were determined by air-drying sediment and sieving it to 2 mm before determination by combustion (Thermo Fisher Scientific Flash Smart Elemental Analyzer, Bremen, Germany). Mehlich-3 and a three-acid system were used to extract the TP and TK, respectively. An ICP-AES Optima 8000 instrument (Perkin-Elmer, Waltham, MA, USA) was used to measure the contents of TP and TK. In addition to the soil measurements, the latitude, longitude, and elevation of each sampling site were recorded in the fields. Mean annual temperature (MAT) and mean annual precipitation (MAP) were obtained from the WorldClim (www.worldclim.org, version-2), and the salinity of each site was obtained from the TPDC database (https://data.tpdc.ac.cn/). The criteria of stress classification for these alpine wetlands were based on the method of Liu et al. [24].

### 2.3 Molecular analyses

Total DNA was extracted from soil samples using the FastDNA Spin Kit for Soil (MoBio Laboratories Inc., Carlsbad, CA, USA), following the manufacturer’s protocol. The amount and quality of the extracted DNA were measured using a NanoDrop 2000 UV-Vis spectrophotometer (Thermo Fisher, Wilmington, MA, USA). The V4-V5 hyper-variable regions of bacterial 16S rRNA gene were PCR amplified using the primer set of F515: 5’-GTGCCAGCMGCCGCGG-3’, R907: 5’-CCGTCAATTCMTTTRAGTTT-3’ [28]. The V3-V5 hypervariable regions of archaeal 16S rRNA gene were PCR amplified using the primer set of Arch344F: 5’-ACGGGGYGCAGCAGGCGCGA-3’, Arch915R: 5’-GTGCTCCCCCGCCAATTCCT-3’ [29]. The PCR reaction was conducted in triplicate with a program of 95 °C for 3 minutes followed by 29 cycles of 95 °C for 30 seconds, 53 °C for 30 seconds, and 72 °C for 45 seconds, ending with a final extension at 72 °C for 10 minutes. The amplicons were sequenced using the Illumina MiSeq platform PE300 (Illumina Inc., San Diego, CA, USA) and have been submitted to the Sequence Read Archive (SRA) under BioProject accessions PRJNA787653 for archaea and PRJNA787654 for bacteria.

The raw sequences were subjected to quality control, clustering, and annotation as follows: First, we merged the paired-end reads using FLASH [30]. Cutadapt software (version 1.9.1) was utilized for quality filtering and trimming [31]. This involved truncating merged reads with a quality score less than 30 and shorter than 200 base pairs (bp), removing amplifying primers, and identifying adaptors. Putative chimeric sequences were then removed by performing a combination of de novo and reference-based chimera checking using Vsearch [32]. The remaining sequences were denoised and dereplicated into Amplicon Sequencing Variants (ASVs, also called phylotypes for convenience) at a 2% maximum expected error threshold, using the DADA2 algorithm in QIIME2 [33]. Taxonomy was assigned to each ASV through the use of the sklearn-classifier with a pre-fitted taxonomy classifier that was specifically trained on the amplified bacterial and archaeal regions [34]. The reference database for bacterial and archaeal taxonomy was the SILVA v.132 release (https://www.arb-silva.de) in the QIIME2 platform.

After a series of quality controls, we retained 9,162,676 bacterial sequences assigned to 45,177 ASVs and 7,234,356 archaeal sequences assigned to 6,308 ASVs. To ensure a consistent sequencing depth, the bacterial dataset was subsampled to 10,335 sequences and the archaeal dataset to 15,968 sequences (i.e., the minimum number of sequences among 200 samples). This resulted in the observation of 13,566 bacterial ASVs and 1,087 archaeal ASVs in the alpine wetlands. The rarefied ASV abundance tables were used to analyze the quantification of microbial diversity.

### 2.4 Inference and quantification of ecological networks

We used a general pipeline to infer and quantify the network associations of bacteria and archaea at the community level (QCMI, https://github.com/joshualiuxu/qcmi) [35]. The process involved four steps. First, we inferred ecological networks using the Spiec-Easi method based on the rarefied ASV abundance table [36]. We then filtered the abundance table with two constraints, retaining only ASVs that appeared in more than 20% of samples and those with sequence abundances greater than 100 to improve the reliability and robustness of the network. This resulted in 3,195 bacterial ASVs and 848 archaeal ASVs used to infer ecological networks. We classified significantly linked pairs of ASVs as either mutual exclusion links (negative relationships) or co-presence links (positive relationships). The Spiec-Easi method was able to identify the weakly connected but real relationships. The criteria for splitting sub-networks were based on whether phylotypes (i.e., ASVs) stably exist in different types of habitats. Subsequently, sub-networks were derived by retaining phylotypes within each subgroup that are deemed reliable in the overarching network. The selection threshold for this study involved controlling for the occurrence (> 30% of all samples within a specific habitat type) and abundance (with mean abundances > 100 reads) of phylotypes in subsets of relative abundance tables corresponding to different wetland types (i.e., freshwater, brackish and saline) [37].

Second, we used sequential correlations to assign assembly processes to significant network associations calculated by spatial distance and environmental dissimilarity [18]. We determined whether the relative abundance of a pair of ASVs was correlated with geographic distance or environmental dissimilarity and categorized the process-based relationship accordingly. The relationships that were unrelated to space and environments were considered putative biotic associations. The filtered biotic associations will be put in the next step.

Third, we manipulated the result tables from the previous steps using the tidyverse package and calculated important network topologies, such as network modularity and fragility, using the ggClusterNet package.

Finally, we averaged the positive and negative correlation strengths among pairs at the ASV level, defined as phylotype connectedness. This step avoided the misunderstanding of many weak associations at the pairwise level by weighting relative abundance with correlation strengths to the community level. Then, we obtained the community complexity of positive and negative associations (thus called positive and negative cohesion) by multiplying the abundance of corresponding phylotypes with the positive and negative connectedness [38]. Network cohesion integrates the connectivity and relative abundance of network nodes, representing both topology and abundance aspects, thereby making it a comprehensive metric for assessing network complexity in microbial ecology. Thus, we used the cohesion metrics in subsequent analyses representing the strength of potential cooperation and competition for bacteria and archaea at the community level.

### 2.5 Statistical analyses

Bacterial and archaeal diversity was assessed using species richness (number of phenotypes), and the Least Significant Difference (LSD) test determined significant differences across salinity levels. To depict the intricacy of ecological networks in these microbial communities, we analyzed network cohesion, encompassing both positive and negative aspects. The standardized effect size (Cohen’s *d*) was calculated to evaluate the differences in network cohesion between the different salinity levels, offering a clear understanding of the practical significance and avoiding the assumptions of statistical tests (e.g., t-test). The magnitude of the effect was defined as large (|*d*| > 0.8, ***), medium (0.5 < |*d*| ≤ 0.8, **), small (0.2 < |*d*| ≤ 0.5, *), and negligible (|*d*| ≤ 0.2). Both linear (Ordinary Least Squares Regression, OLS) and non-linear (General Additive Model, GAM) methods were utilized to analyze the relationship between stress and microbial response, with the Akaike Information Criterion (AIC) guiding model selection. We addressed multicollinearity among variables using the Variance Inflation Factor (VIF).

To analyze the community composition of bacteria and archaea, the rarefied ASV table was first Hellinger transformed, and then Bray–Curtis dissimilarity was calculated using the vegan package. Non-metric Multidimensional Scaling (NMDS) was performed to determine the ordination patterns of bacterial and archaeal communities, displayed as a three-dimensional plot using the vegan package. Complementary nonparametric analyses, including ADONIS, ANOSIM, and MRPP, were conducted to assess the dissimilarity in community composition across varying salinity levels using the microeco package.

Structural Equation Modeling (SEM) was applied to discern the direct and indirect influences of factors such as soil, climate, space, stress, and ecological networks on microbial diversity. A Random Forest (RF) model initially identified key factors impacting microbial diversity, informing variable selection for SEM. These variables represented latent variables associated with soil, climate, space, and ecological networks. The piecewiseSEM package in *R*, incorporating nlme and lme4, facilitated this analysis, allowing for a comprehensive exploration of the intricate relationships among abiotic and biotic variables and diversity under stress [39]. The piecewiseSEM accounts for the random effects of sampling plots by providing marginal and conditional contributions of predictors. We confirmed the goodness of the modeling results using Fisher’s *C* test (when 0 < Fisher’s *C*/d.f. < 2 and *P* > 0.05).

## 3 Results

### 3.1 Diversity of bacteria and archaea across different salt stress levels

**Fig. 2:**
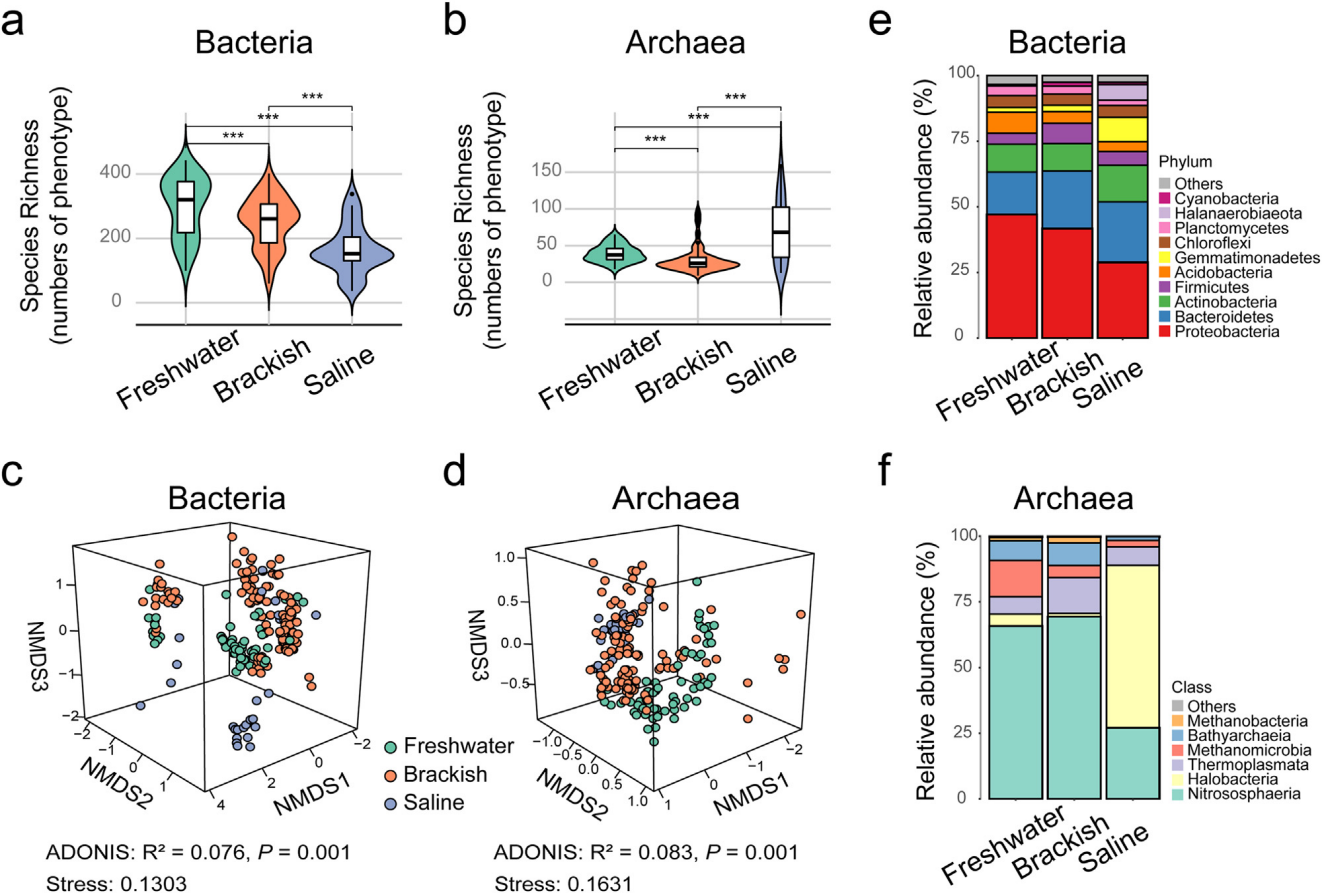
**Diversity and community composition of bacteria and archaea in alpine wetlands of the Tibetan Plateau.** (a,b), Comparisons of bacterial and archaeal diversity (numbers of phenotypes) across freshwater, brackish, and saline wetlands. The results of Least Significant Difference (LSD) test were shown the among-group comparison in diagrams, with *** denoting *P* < 0.001. (c,d), Three-dimensional ordination using NMDS shows the differentiation in community composition of bacteria and archaea among freshwater, brackish, and saline lake wetlands. The results of ADONIS are shown in diagrams. (e,f), Taxonomic composition of bacterial and archaeal communities across freshwater, brackish, and saline wetlands. The results are shown at the bacterial phylum and archaeal class levels, respectively.

We initiated the investigation by assessing the species richness of bacterial and archaeal communities across 20 alpine wetlands (Fig. 1). Our analysis unveiled marked variation patterns in both bacterial and archaeal richness. Specifically, bacterial diversity exhibited a discernible decline in tandem with escalating salinity levels, following a gradient from low to high-stress conditions (Fig. 2a). In contrast, archaeal diversity peaked in saline wetlands while reaching its nadir in brackish environments, thereby illustrating a high-stress to low-stress to the medium-stress pattern (Fig. 2b). These trends were corroborated by supplementary diversity indices (Table S2). Furthermore, the trajectory of diversity shifts across each wetland field closely mirrored the diversity trends observed across the types of wetlands (Fig. S1). In both instances, bacterial diversity waned with increasing salinity, while archaeal diversity initially decreased before rebounding.

We then enumerated the number of overlapping Amplicon Sequence Variants (ASVs, also called phylotypes) of bacteria and archaea across the alpine wetlands with different stress levels (Fig. S2). The results indicated that 32.9% of the bacterial ASVs and 56.8% of the archaeal ASVs were common to the three types of wetlands. A high proportion of ASVs was shared between freshwater and brackish wetlands, with 65.0% for bacteria and 79.2% for archaea, suggesting a similar composition of phylotypes between low and medium salinity stress. However, the proportion of exclusively shared ASVs between freshwater and saline wetlands was relatively low (7.8% for bacteria and 2.0% for archaea), indicating a strong difference in their community composition between low and high-stress wetlands.

The results of NMDS found that the dissimilarity of bacterial and archaeal communities was affected by the stress levels and differed across freshwater, brackish, and saline wetlands (Fig. 2c–d; Table S3). The taxonomic composition of bacterial communities was dominated by Proteobacteria, Bacteroidetes, and Actinobacteria, accounting for more than 50% of total sequences across the three types of wetlands, consistent with previous findings (Figs. 2e and S3, S4; Table S4) [40]. In terms of archaeal communities, Nitrososphaeria (Thaumarchaeota phylum) was the predominant class in freshwater (∼66%) and brackish (∼69%) wetlands, while Halobacteria (Euryarchaeota phylum) dominated in saline wetlands (∼62%) (Figs. 2f and S3, S4; Table S4).

**Fig. 3:**
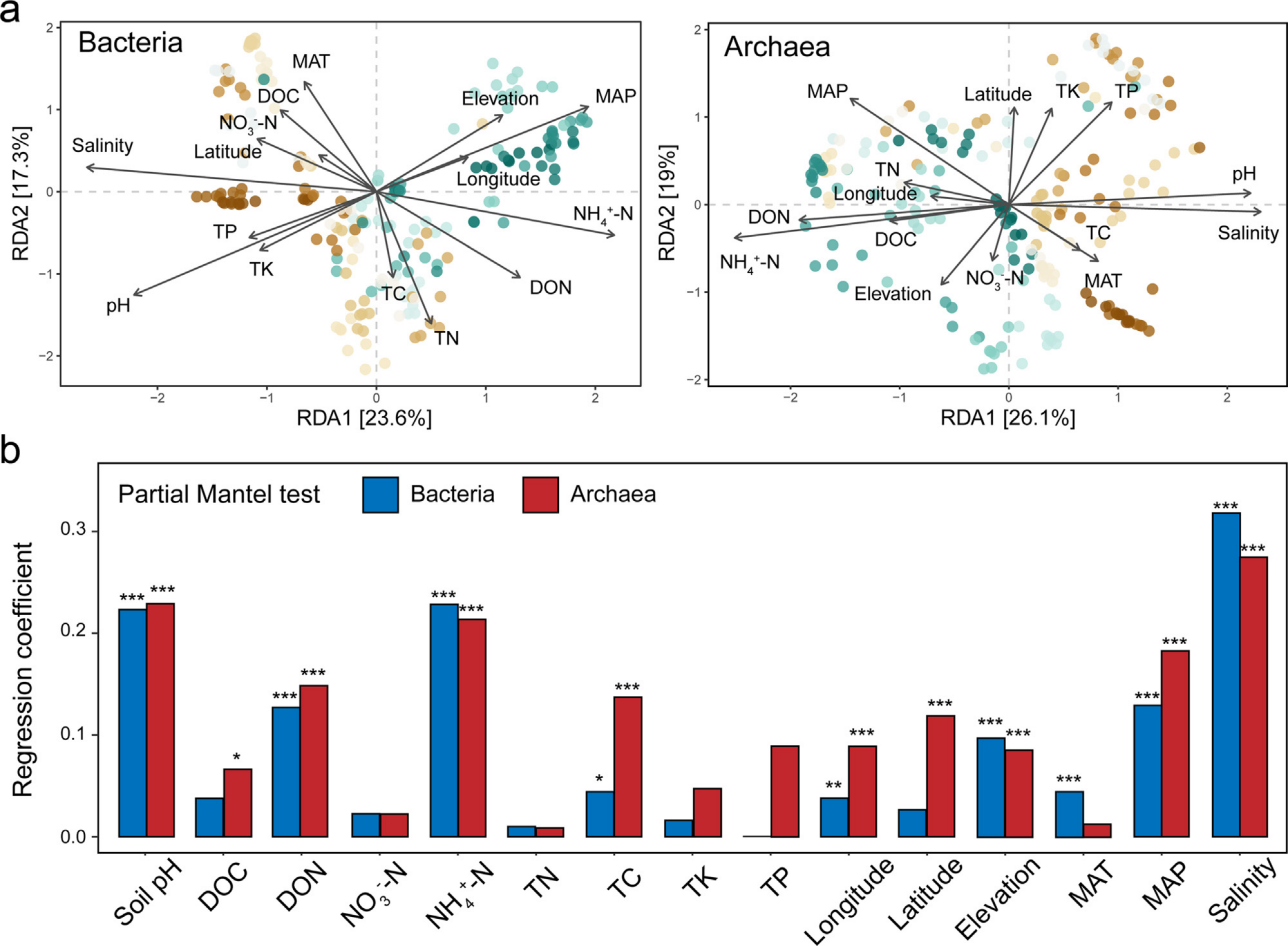
**Effects of environmental variables on bacterial and archaeal communities in the alpine wetlands of the Tibetan Plateau.** (a) Distance-based redundancy analysis (dbRDA) ordination plots of the community-environment relationships of bacterial and archaeal communities, as measured by Bray-Curtis distance. (b) Community composition (measured using Bray-Curtis distance) is examined in relation to different factors including soil properties, climate, spatial variables, and salinity, individually. Asterisks denote statistical significance, validated through 999 permutations in Mantel tests.

Furthermore, we delved into what variables drive the variations in bacterial and archaeal communities (Fig. 3). Utilizing the dbRDA method with forward selection, we found that almost all tested factors, except NO_3_^−^-N, significantly impacted the bacterial community structure. Notably, two factors stood out: salinity (*R*^2^ = 0.54, *P* = 0.001) and soil pH (*R*^2^ = 0.48, *P* = 0.001), which were crucial in shaping bacterial communities within alpine wetlands. In contrast, for archaeal communities, NH_4_^+^-N (*R*^2^ = 0.45, *P* = 0.001) and salinity (*R*^2^ = 0.37, *P* = 0.001) emerged as the primary influencing factors. Confirming these findings, partial Mantel tests, which accounted for other variables, consistently highlighted salinity'’s pivotal role in the variation observed in both bacterial and archaeal communities.

### 3.2 Ecological networks of bacteria and archaea across different salt stress levels

**Fig. 4:**
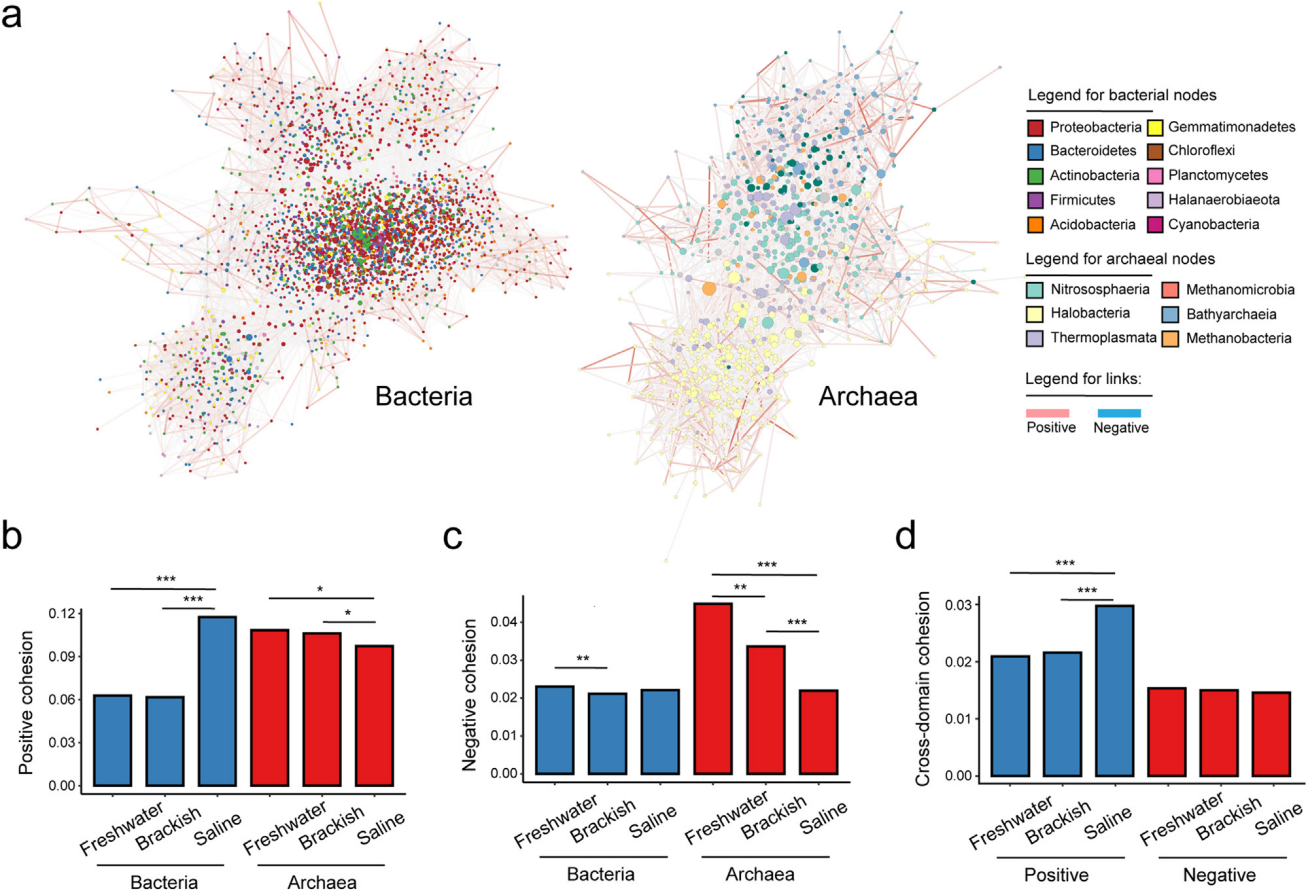
**Ecological networks of bacteria and archaea in alpine wetlands of the Tibetan Plateau.** a, Ecological networks were shown by Cytoscape for bacteria and archaea, respectively. Colors of nodes indicated major taxa. Pink links represented positive associations between pairs of phylotypes. Light blue links represented negative associations between pairs of phylotypes. b–c, Positive and negative cohesion of bacteria (blue) and archaea (red) between alpine wetlands on behalf of the community complexity of network associations. d, Positive and negative cohesion for cross-domain associations between bacteria and archaea across different types of alpine wetlands. Asterisk symbol represented large (|*d*| > 0.8, ***), medium (0.5 < |*d*| ≤ 0.8, **), and small (0.2 < |*d*| ≤ 0.5, *) effect sizes, based on Cohen’s *d* (the mean difference divided by pooled standard deviation).

We established ecological networks for bacterial and archaeal taxa, encompassing 3,195 and 848 phylotypes, respectively, after processing the rarefied ASV tables (Fig. 4a). Within these networks, nodes of differing colors represent various taxa. The bacterial network consisted of 87,988 links, with negative relationships accounting for 36.5% of these. Meanwhile, the archaeal network had 6,706 links, with negative associations comprising 16.2%. These networks were then evaluated under different levels of salinity stress conditions (Table S5). To delve deeper, we isolated three sub-networks for both bacterial and archaeal communities, each representing a distinct stress level, and calculated their topological properties (Fig. S5). Our analysis of these sub-network topologies revealed significant variations in response to salinity levels (Tables S5, S6). Specifically, we assessed network fragility, defined as the change in natural community connectivity due to the loss of key taxa under simulated stress conditions, to gauge network stability (Fig. S6). Our findings indicated that bacterial networks became increasingly fragile, while archaeal networks exhibited enhanced resilience as stress levels escalated. Although we evaluated multiple metrics to measure network complexity, no consistent pattern was discernible (Table S5).

To further elucidate the complexity of bacterial and archaeal ecological networks, we quantified network cohesion [38]. Our analysis showed that positive cohesion for archaea was diminished in saline wetlands compared to freshwater and brackish environments. Conversely, positive cohesion for bacteria was elevated in saline wetlands (Fig. 4b). Additionally, negative cohesion in archaeal networks decreased with increasing stress, a more significant shift than observed in positive cohesion. In comparison, bacterial networks showed no clear trend in negative cohesion (Fig. 4c). Further analysis from individual wetlands showed that bacterial cohesion increased significantly under high salt stress. In contrast, archaeal cohesion generally decreased with increasing stress for both positive and negative associations (Fig. S7). These findings illuminate the potentially contrasting strategies that bacteria and archaea may employ to adapt to salinity stress.

In addition, we examined the cross-domain relationships between bacteria and archaea. We broadened our scope to include these cross-domain relationships, weighting them by relative abundance to calculate a measure of cross-domain cohesion. This encompassed both positive and negative associations (Fig. 4d). We found that significant differences existed only in the positive cross-domain cohesion of saline wetlands compared to those of freshwater and brackish wetlands.

### 3.3 Responses of diversity and ecological networks along the salt stress gradient

**Fig. 5:**
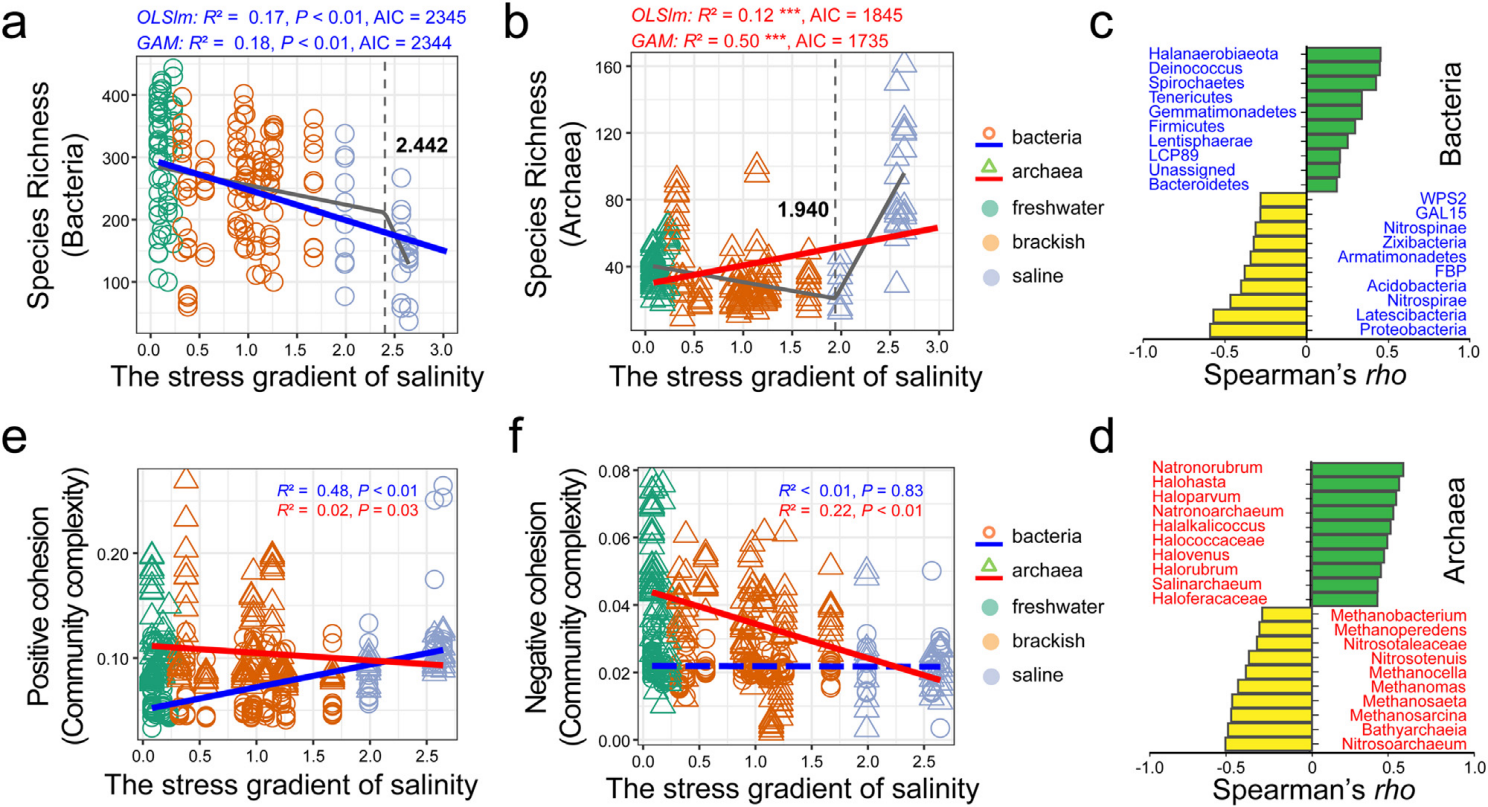
**The responses of diversity and ecological networks of bacteria and archaea to the stress gradient.** a–b, The relationships between the stress gradient (salinity, psu) and diversity (species richness, numbers of phylotypes) for bacteria and archaea, respectively. The gray piecewise line indicated the non-linear relationships and the straight line indicated the linear relationships. The circle and triangle points represented bacteria and archaea in freshwater (green, low stress), brackish (orange, medium stress) and saline (purple, high stress) wetlands. Statistics were also attached in the diagrams, corresponding to the *R*^2^ value and *P* significance. c–d, Major taxa of bacteria and archaea most positively and negatively impacted by the salinity stress. Top ten taxa whose abundances were most positively or negatively correlated with the stress gradient. The *P* values were adjusted by false discovery rate. e–f, The relationships between the stress gradient (salinity, psu) and community complexity of positive and negative associations (network cohesions) for bacteria and archaea. The attached information was consistent with diagram a and b.

We sought to explore both linear and non-linear relationships between salinity and microbial diversity, as well as ecological networks within the 20 alpine wetlands. First, we delineated the linear relationships of diversity across an extensive salinity stress gradient (Fig. 5a–b). Our findings indicated a negative correlation between bacterial richness and salinity (*R*^2^ = 0.17, *P* < 0.01), while archaeal richness positively correlated with salinity (*R*^2^ = 0.12, *P* < 0.01). We further employed non-linear regression analyses, as measured by Generalized Additive Models (GAM), to identify stress thresholds impacting the diversity of bacterial and archaeal communities. Intriguingly, both bacterial and archaeal communities exhibited significant stress thresholds characterized by abrupt changes in slope magnitude. Specifically, bacterial taxonomic richness experienced a sudden decline when salinity surpassed a stress threshold of approximately 2.44. Conversely, archaeal richness initially decreased up to a salinity unit of 1.94 and then sharply increased. A comparative analysis between linear and non-linear models revealed negligible differences in goodness-of-fit (i.e., AIC) for bacterial diversity, whereas the non-linear model was superior for archaeal diversity, boasting an explanatory rate as high as ∼50%.

Furthermore, we employed correlation models to identify the bacterial and archaeal taxa most significantly influenced by salinity variations (Fig. 5c–d). Our analysis revealed that archaeal taxa, such as Natronorubrum and Halohasta, positively correlated with salinity and were primarily stress-tolerant. Bacteria responding positively to salinity included both rare soil taxa, like Spirochaetes and Tenericutes, and stress-tolerant taxa, such as Halanaerobiaeota and Deinococcus. In contrast, taxa negatively impacted by salinity were primarily those involved in carbon and nitrogen cycling, including Proteobacteria for bacteria and Nitrosoarchaeum for archaea.

We examined the relationships between the salinity stress and ecological networks (Fig. 5e–f). Linear analyses indicated that bacterial positive cohesion significantly increased with rising salinity (*R*^2^ = 0.23, *P* < 0.01), whereas the correlation between stress and bacterial negative cohesion was not statistically significant (*R*^2^ < 0.01, *P* = 0.83). For archaea, negative cohesion significantly decreased with increasing stress (*R*^2^ = 0.22, *P* < 0.01), while positive cohesion exhibited a slight but significant decline (*R*^2^ = 0.02, *P* = 0.03). Subsequent non-linear analyses (Fig. S8) revealed that bacterial positive cohesion notably increased when salinity reached approximately 1.77 units, whereas negative cohesion plateaued around a salinity unit of 0.18. For archaea, positive cohesion showed a slight increase around a salinity unit of 2.43, while negative cohesion consistently decreased without any detectable threshold. In addition, the change point was only observed in the relationship between positive cross-domain cohesion and salinity stress, around a unit of ∼1.50, yet with no threshold identified for negative cross-domain cohesion.

### 3.4 Diversity associated with environmental variables and ecological networks

**Fig. 6:**
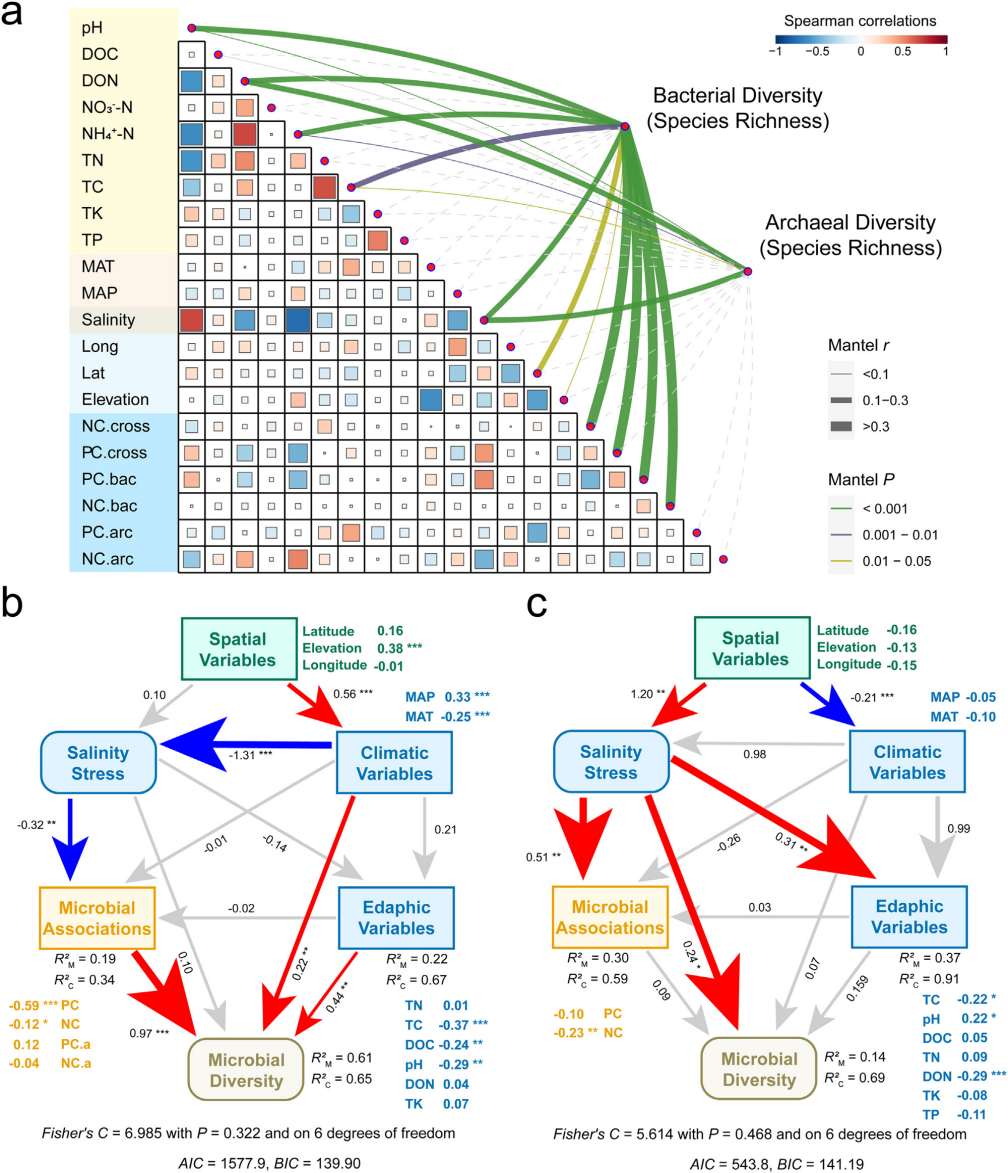
**The driving forces of bacterial and archaeal diversity associated with environmental variables and ecological networks.** (a) Pairwise comparisons of explained variables, including soil, climate, space, salinity and ecological networks, were shown with a color gradient denoting Spearman’s rank correlation coefficients. Correlations between microbial diversity and explained variables were shown by curve lines. The line width denotes the correlation coefficient determined by the linear mixed-effects model (sampling plots as random factors). Statistical significance is based on Wald type II χ² tests with n = 200 independent samples. The *P* values were adjusted by false discovery rate; ***P < 0.001, **P < 0.01, *P < 0.05. PiecewiseSEM accounting for the direct and indirect effects of multiple variables (space, climate, soil properties, salinity and ecological networks) on biodiversity (species richness) of bacteria (b) and archaea (c) across all alpine wetlands of the Tibetan Plateau. Space, climate, soil properties, and ecological networks were composite variables. Numbers adjacent to the measured variables were their coefficients with composite variables. Numbers adjacent to arrows were path coefficients and were the directly standardized effect size of the relationship. The thickness of the arrow represented the strength of the relationship. Abbreviations for variable were shown in the Supporting Information. The conditional and marginal *R*^2^ represent the proportion of variance explained by all predictors without and with accounting for random effects of “sampling plots”. Significance levels of each predictor are * *P* < 0.05, ** *P* < 0.01 and *** *P* < 0.001. Red and blue arrows indicated significantly positive and negative relationships, respectively. The insignificant relationships were indicated by grey lines. Fisher’s C with *P*-value was the goodness-of-fit statistics for each model. All explanatory variables were pre-detected by random forest for the explanation of diversity, and the feeble variables with *P* > 0.05 in the RF model were not included in the SEM.

Finally, we aimed to elucidate the mechanisms underpinning the diversity of bacteria and archaea in relation to environmental variables and ecological networks within such stressful habitats. To achieve this, we employed a dual-method approach, incorporating both ordinary least square regression (OLS) and random forest (RF) models, to scrutinize the relationships between microbial diversity, environmental factors, and ecological networks (Figs. S9-11; Table S7). Our findings revealed that bacterial diversity was most strongly associated with positive cohesion, although other variables such as DOC, NH_4_^+^-N, TC, MAT, MAP, elevation, negative cohesion, and salinity also contributed significantly. Differently archaeal diversity was predominantly influenced by abiotic factors, with salinity emerging as the most critical. Soil pH and nitrogen levels also demonstrated high relative importance. We further employed linear mixed-effects models (LMM), treating sampling plots as random factors, to investigate bacterial and archaeal diversity. Our analysis indicated that different microbial groups exhibited varied responses to ecological networks and environmental variables (Fig. 6a; Table S8), consistent with the above analyses, suggesting that microbial networks offer new perspectives on the maintenance of microbial biodiversity.

To gain a more nuanced and detailed understanding, we employed SEM to break down the direct and indirect effects of environmental variables and ecological networks on bacterial and archaeal diversity (Fig. 6b–c). Based on the results from the Random Forest (RF) model, SEM analysis verified that positive cohesion was the strongest predictor of bacterial diversity. In contrast, salinity emerged as the most significant factor shaping archaeal diversity, even when other environmental and ecological variables were considered (Fig. S12; Table S9). For the composite model of bacterial communities, spatial factors directly influenced climatic variables, which in turn significantly accounted for variations in salinity, which significantly affected bacterial network complexity in response to stress (Fig. 6b). A synergy between climatic and soil variables, as well as microbial associations, supported bacterial biodiversity. For archaea, salinity was the most influential factor, impacting not only archaeal association complexity but also soil properties and, ultimately, archaeal biodiversity (Fig. 6c).

## 4 Discussion

The first aim in this study was to investigate the relationship between salt stress and the diversity of bacteria and archaea in alpine wetlands on the Tibetan Plateau. Our results showed that salinity stress has a significant but different relationship with the diversity of bacteria and archaea (Fig. 2, 5a–b). In accordance with previous studies [6,^7^,25], bacterial diversity decreased as the salinity gradient increased. This decrease can be attributed to the accumulation of salt in the soil, which raises the extracellular osmolarity, causing many bacterial taxa to become inactive or die, thus reducing the diversity [41]. Interestingly, archaeal diversity exhibits a non-linear response to increasing salinity stress, characterized initially by a decrease, followed by a subsequent increase. Combined with the result of archaeal taxa, the pattern indicates that under low salinity conditions, the diversity reduction is largely due to the vulnerability of certain stress-sensitive taxa, such as ammonia-oxidizing archaea and methanogens (Figs. 5 and S4). These groups are often the first affected, leading to a decline in overall species diversity. However, as salinity stress escalates, more resilient, stress-tolerant taxa, such as halophilic archaea, begin to proliferate [6,[41], [42], [43]. This trend prompts a broader exploration of the adaptive strategies of archaeal communities in response to stress. The initial reduction in diversity represents a phase of ecological adjustment, where more sensitive species diminish or are outcompeted. Subsequently, a phase of resilience emerges, characterized by the dominance of species better equipped for high salinity. This transition not only recuperates diversity but may also establish a new ecological balance, forming a community more attuned to these conditions. These dynamics highlight the intricate relationship between stress factors and microbial community resilience, underscoring the imperative for further research to unravel the mechanisms behind these changes [44], [45], [46].

Moreover, our study also highlights a significant gap in understanding the diversity response thresholds of microbial taxa along the salinity gradient. Recognizing these thresholds is vital for predicting ecosystem responses to global changes, as they mark critical points where minor variations in salinity can lead to major ecological shifts. Focusing on integrating these thresholds into broader ecological models may enhance our ability to anticipate and mitigate the impacts of environmental stressors on microbial communities and ecosystem functions.

The most fundamental issue concerned in this study was how the ecological networks of bacteria and archaea respond to stress gradients in order to understand their coping strategies at the community level. Our results revealed that the positive cohesion of bacteria increased with increasing stress, while negative cohesion remained unchanged (Fig. 5e–f). This supports the general prediction of the stress gradient hypothesis for the core hypothesis [17,^26^,47]. We believe that bacteria increase their potential for cooperation in response to stress to access new or diverse resources and to ensure survival and that cooperation allows certain taxa to expand their niche and survive under stress [9,48]. Therefore, we refer to this strategy as the “associational resistance” of bacteria in response to increasing stress.

However, the changes in the ecological networks of archaea were unexpected, with a slight decrease in positive cohesion and a drastic decrease in negative cohesion. We suggest that this result is linked to the changes in the major taxa of archaea along the stress gradient. In low-stress environments, such as freshwater and brackish wetlands, the archaeal communities were dominated by functional taxa capable of mediating the carbon and nitrogen cycles, leading to cooperate (e.g., similar processes or functions) or compete (e.g., similar substrates) [49,50], while in high-stress conditions, for example, saline wetlands, the archaeal communities were dominated by stress-tolerant taxa (e.g., halophile), which are relatively independent of each other based on the individual traits (e.g., membrane composition) [9,51]. We investigate facilitation models within ecological theory to elucidate the reinforcement of positive relationships under stress. These models encompass several aspects: the integration of facilitation into niche theory, the evaluation of its impact on density-dependent hypotheses, the assessment of its influence on community invasiveness, and the analysis of its effects on community resilience in the face of disturbances. This exploration prompts us to consider various mechanisms potentially driving microbes to amplify their positive associations under stressful conditions. A notable hypothesis is the expansion of ecological niches at the community level through cooperative interactions, presenting a plausible strategy for stress resistance in current environmental scenarios. This perspective implies that facilitation not only bolsters microbial resilience but also plays a crucial role in enhancing the overall adaptability of communities to environmental challenges. Additionally, we performed the initial exploration into the cross-domain network associations between bacteria and archaea. The results further substantiated our findings that the positive relationships between these two domains intensify under increased stress conditions. Thus, we correspondingly propose that archaea adopt the strategy of “trait-based individual resistance” in coping with stress rather than the “associational resistance” seen in bacteria.

Besides, the stress gradient hypothesis has driven numerous studies in recent decades but lacks a unified framework to describe microbial interactions [19,^26^,52]. Our findings provide empirical evidence about the patterns of ecological networks for bacteria and archaea along the salt stress gradient, potentially helping integrate microbial traits into classical ecology theory and improving predictions and interpretations of outcomes.

Recent studies have predominantly explored the relationships between environmental variables and microbial diversity, unveiling that variations in microbial diversity are predominantly governed by the environmental selection aspect of the assembly process [17,^18^,43]. However, the intricate mechanisms underpinning the maintenance of microbial diversity under adverse conditions remain poorly understood, attributed to the dearth of studies on ecological associations among microbes, hinting at the role of biotic filtering [27]. Our findings illuminate the significant explanatory and predictive power of positive cohesion for bacterial diversity, whereas alterations in salinity predominantly influence archaeal diversity (Fig. 6). Previous research has underscored the vital influence of negative interactions among microbes on community structure and functionality [15,16], with experiments involving the deliberate removal of populations from natural soil microbial communities revealing that competitive dynamics significantly impact the re-colonization process by dominant bacterial taxa, thereby highlighting the critical role of negative associations in community assembly [49]. Our investigation, anchored in empirical data, reveals an escalation in the complexity of positive associations among bacteria in response to increasing stress, indicating a mechanism of resilience to environmental pressures through enhanced cooperative potential. For archaea, our findings from structural equation modeling, coupled with observations of stress-induced shifts in major taxa, indicate that the predominance of salt-tolerant taxa is a key driver of changes in archaeal diversity under escalating stress conditions (Figs. 5 and 6). These insights posit biotic filtering as a pivotal mechanism in stabilizing bacterial diversity and safeguarding vital functions under stress, while environmental selection is identified as the main determinant of archaeal diversity [53], [54], [55]. Moreover, the traditional focus of soil conservation has been on preserving soil biodiversity, community heterogeneity, and ecosystem services. However, our study suggests that microbial associations should also be considered in microbial conservation strategies, underscoring their significance in sustaining biodiversity and regulating ecosystem services [5].

## 5 Conclusion

In summary, our research offers important empirical insights into the diversity and ecological networks of bacteria and archaea in the context of varying salinity levels in the alpine wetlands of the Tibetan Plateau. We found marked adaptive disparities between the two prokaryotic groups under salt stress. Specifically, bacterial diversity showed a downward trend as salinity increased, while archaeal diversity exhibited a non-linear response. Furthermore, network analyses revealed that stress conditions led to an enhanced complexity of positive associations among bacterial communities, while both positive and negative associations weakened among archaea. These trends imply that bacteria potentially counter stress through enhanced mutualistic relationships, while archaea likely depend on salt-tolerant taxa for survival in stressful settings. These findings are corroborated by diversity maintenance mechanisms that consider both environmental variables and network associations. Our observations contribute new insights to our understanding of how microbial diversity is maintained under challenging conditions. Overall, this research not only advances our knowledge of the stress responses of bacteria and archaea but also expands our understanding of strategies for soil biodiversity conservation in extreme ecosystems.

## Data and materials availability

The raw data associated with this study will be publicly available upon manuscript acceptance. The pipeline code used in data analysis is deposited at github.com/joshualiuxu/qmci.

## Declaration of competing interest

The authors declare that they have no conflicts of interest in this work.

## Appendix Supplementary materials

Image, application 1

Image, application 2

Image, application 3
