# Supplementary material for: Contrasting patterns for bacteria and archaea in response to salt stress across alpine wetlands of the Tibetan Plateau
Source: Fundam Res. 2024 Mar 7;6(1):313–23. doi: 10.1016/j.fmre.2024.02.010 (PMC12869736; doi:10.1016/j.fmre.2024.02.010)
Supplement: Supplementary file 2 [file mmc2.docx]

**Supplemental information for**

**Contrasting patterns for bacteria and archaea in response to salt stress across alpine wetlands of the Tibetan Plateau**

**Authors:** Xu Liu^1,2^, Manuel Delgado-Baquerizo^3*^, Teng Yang^1,2^, Gui-Feng Gao^1,2^, Yu Shi^4^, Haiyan Chu^1,2*^

**Affiliations:**

^1^ State Key Laboratory of Soil and Sustainable Agriculture, Institute of Soil Science, Chinese Academy of Sciences, Nanjing 210008, China.

^2^ University of Chinese Academy of Sciences, Beijing 100049, China.

^3^Laboratorio de Biodiversidad y Funcionamiento Ecosistémico. Instituto de Recursos Naturales y Agrobiología de Sevilla (IRNAS), CSIC, Av. Reina Mercedes 10, E-41012, Sevilla, Spain.

^4^ State Key Laboratory of Crop Stress Adaptation and Improvement, School of Life Sciences, Henan University, Kaifeng 475004, China.

***Corresponding authors:**

Haiyan Chu^1,2^*, [hychu@issas.ac.cn](mailto:hychu@issas.ac.cn)

Manuel Delgado-Baquerizo^3,4^*, [M.delgado.baquerizo@csic.es](mailto:M.delgado.baquerizo@csic.es)

**This PDF file includes:**

Captions for Tables S1 to S9

Figures S1 to S12

**Table S1 – S9 are shown in different sheets in the “SI.tables.xlsx” file.**

**Table S1.** Sampling information of 20 investigated wetlands in this study, including edaphic factors, geographic factors, climatic factors, chronic energy stress from salinity, and types of wetlands.

**Table S2.** Alpha diversity of bacterial and archaeal communities across freshwater, brackish, and saline wetlands, measured by species richness, Chao1, ACE, Shannon, Simpson, inv-simpson, Fisher, Coverage, and Faith’s phylogenetic diversity.

**Table S3.** Dissimilarities in bacterial and archaeal community composition between freshwater, brackish, and saline lake wetlands as determined by ADONIS, ANOSIM, and MRPP.

**Table S4.** Relative abundance of bacterial phyla and archaeal class across freshwater, brackish, and saline wetlands. Values are means (SD). Values at the same columns followed by different letters differed significantly at *P* < 0.05.

**Table S5.** Summary of the topological parameters of ecological networks of bacteria and archaea across freshwater, brackish, saline wetlands.

**Table S6.** Summary of topological property and taxonomic information of involved nodes in the networks.

**Table S7.** Summary of the best ordinary least squares (OLS) multiple regression models for the effects of abiotic and biotic variables on species richness of bacteria and archaea.

**Table S8.** Correlations between prokaryotes (diversity and major taxa) and explained variables.

**Table S9.** PiecewiseSEM accounting for the direct and indirect effects of multiple variables (space, climate, soil, salinity and ecological associations) on diversity (species richness) of bacteria and archaea across all alpine wetlands of the Tibetan Plateau.

**Figure S1 – S12** are shown below


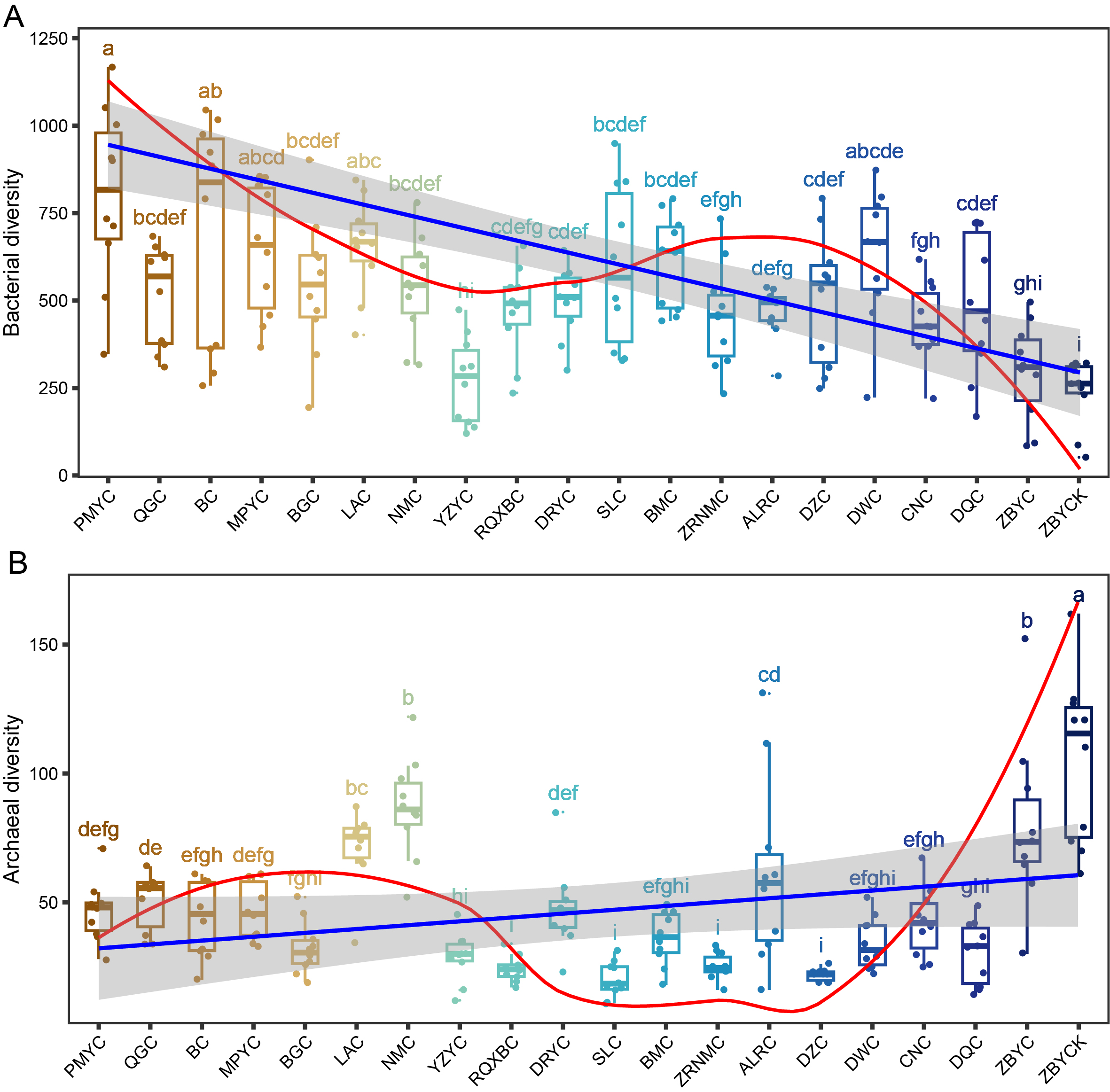


**Fig. S1.** Bacterial and archaeal diversity within each alpine wetland (n = 20). The results of Least Significant Difference (LSD) test were shown the among-group comparison in diagrams, with different letters denoting statistical significance. Statistical analysis for the relationship among site-level diversity was performed using ordinary least squares linear regression (blue line) and loess non-linear regression (red line).

**
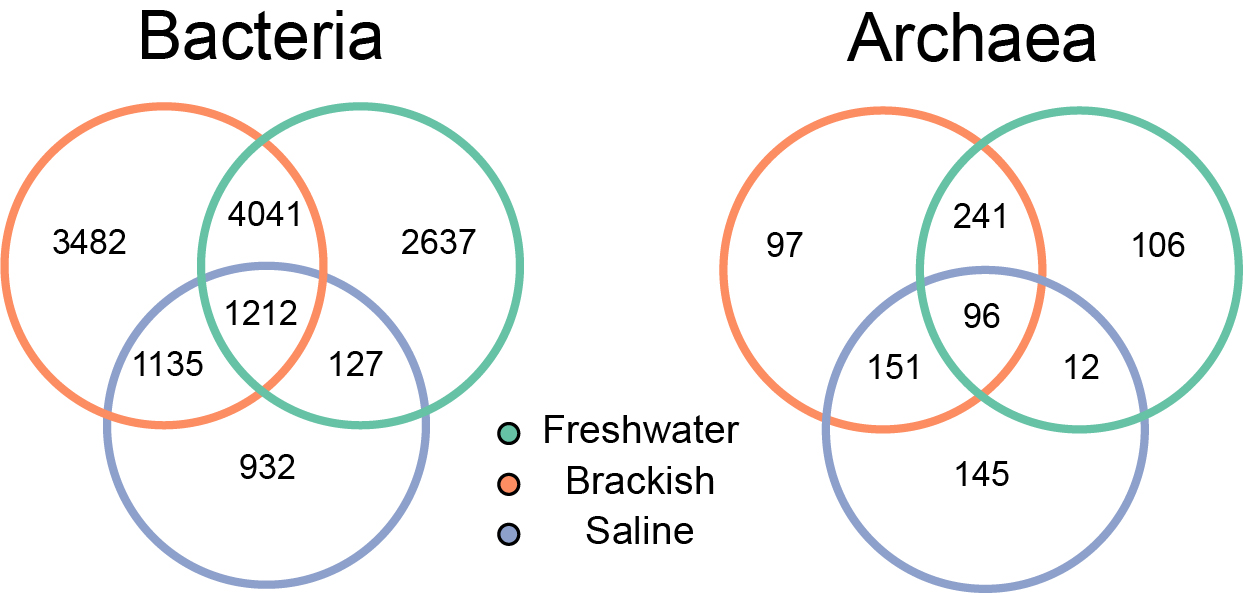
**

**Fig. S2.** Venn diagrams of bacterial and archaeal phenotypes’ distribution among freshwater, brackish, and saline wetlands.


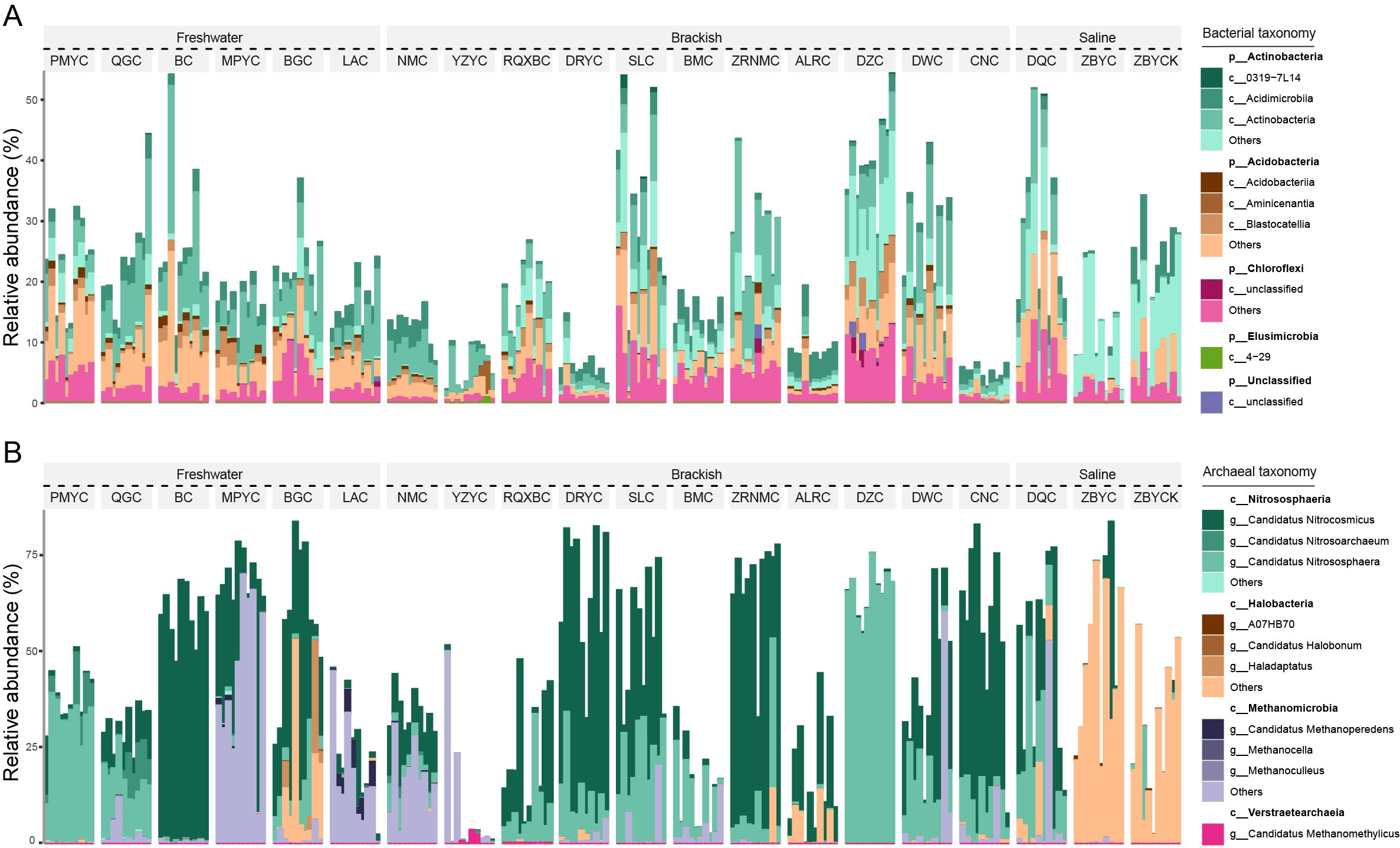


**Fig. S3.** Taxonomic composition of bacterial and archaeal communities across each alpine wetlands. The results are shown at the bacterial class and archaeal genus levels, respectively. The blank areas belong to microbial taxa that were not delineated into the high abundance of the overall sample.


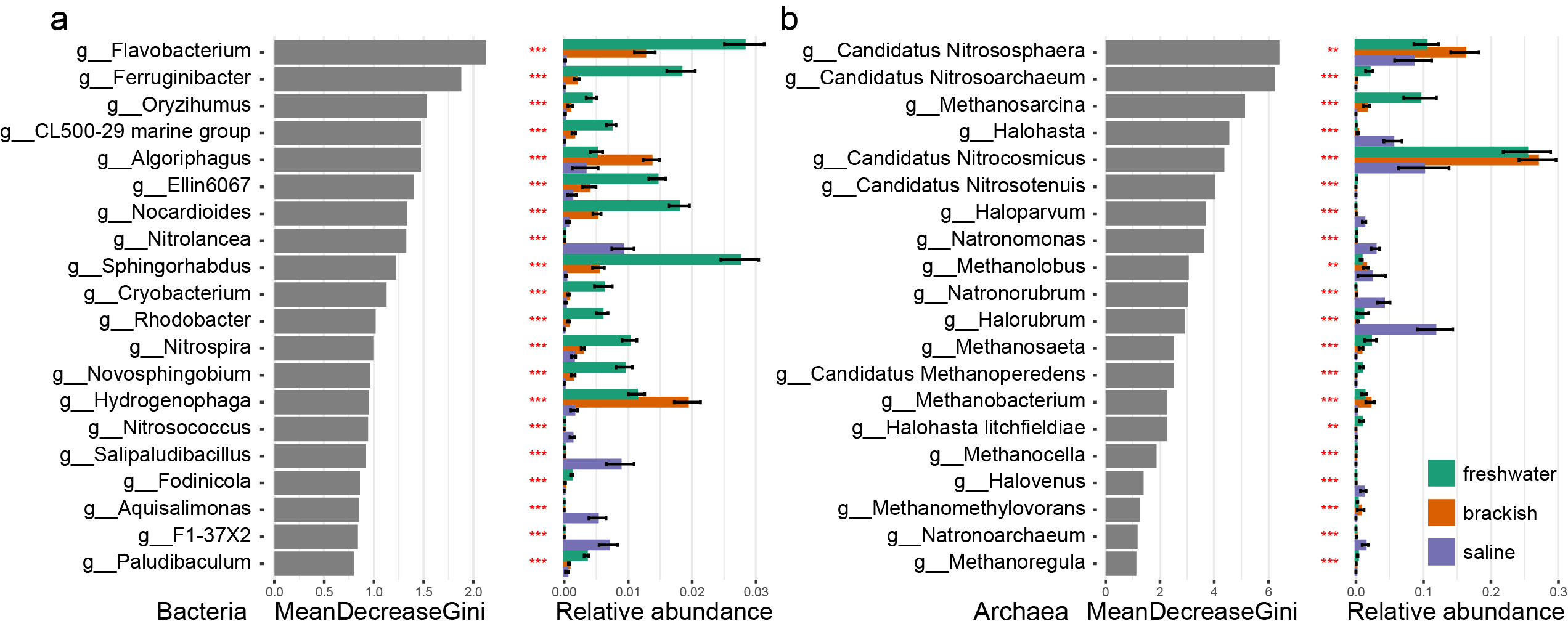


**Fig. S4.** Changes of microbial taxonomic groups among freshwater, brackish and saline wetlands of the Tibetan Plateau. Random-forest model detected the transitions of bacterial **(a)** and archaeal **(b)** taxa across freshwater, brackish and saline wetlands. The top 20 genus were identified, ranked in descending order of importance to the accuracy of the model. *, *P* < 0.05; **, *P* < 0.01; ***, *P* < 0.001.


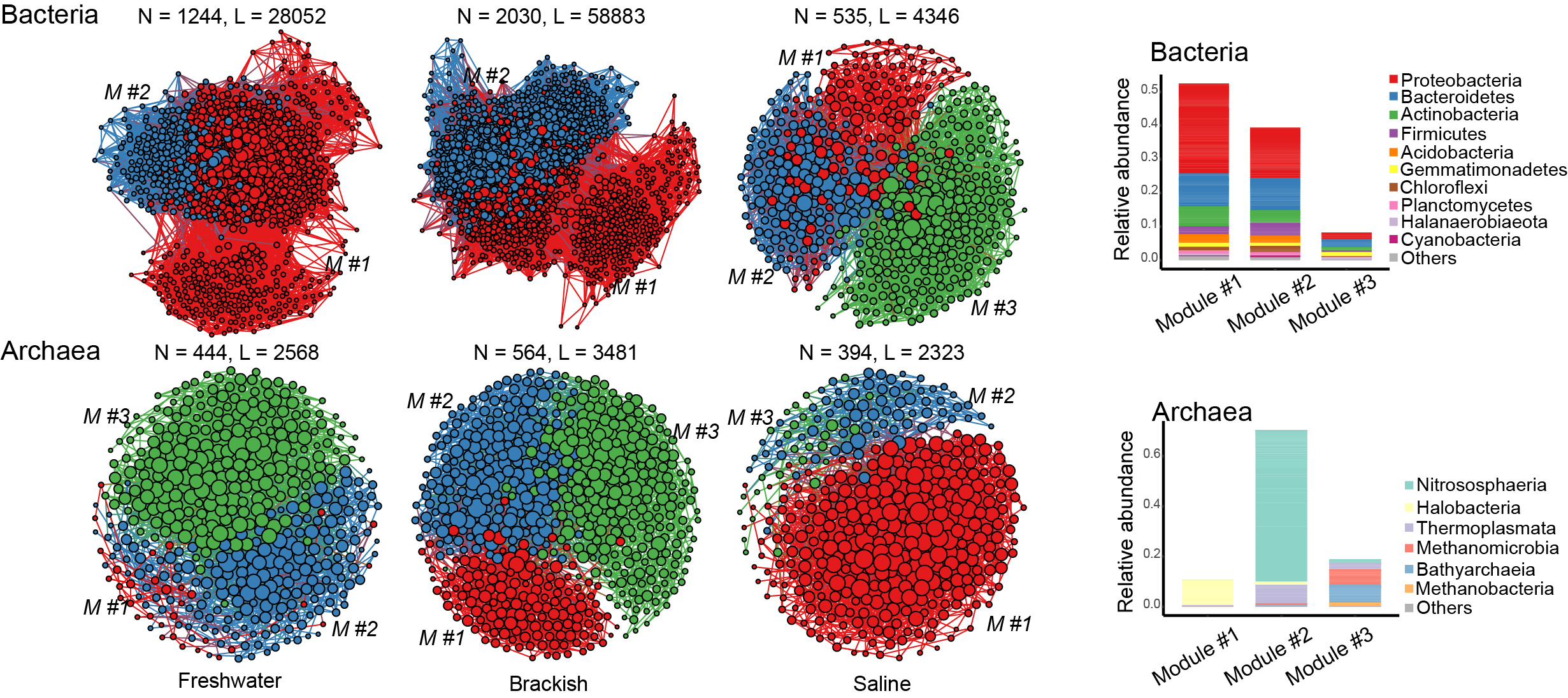


**Fig. S5.** Relative abundance of bacterial and archaeal sub-communities within three modules of ecological networks at the phylum levels and at the class levels, respectively.

**
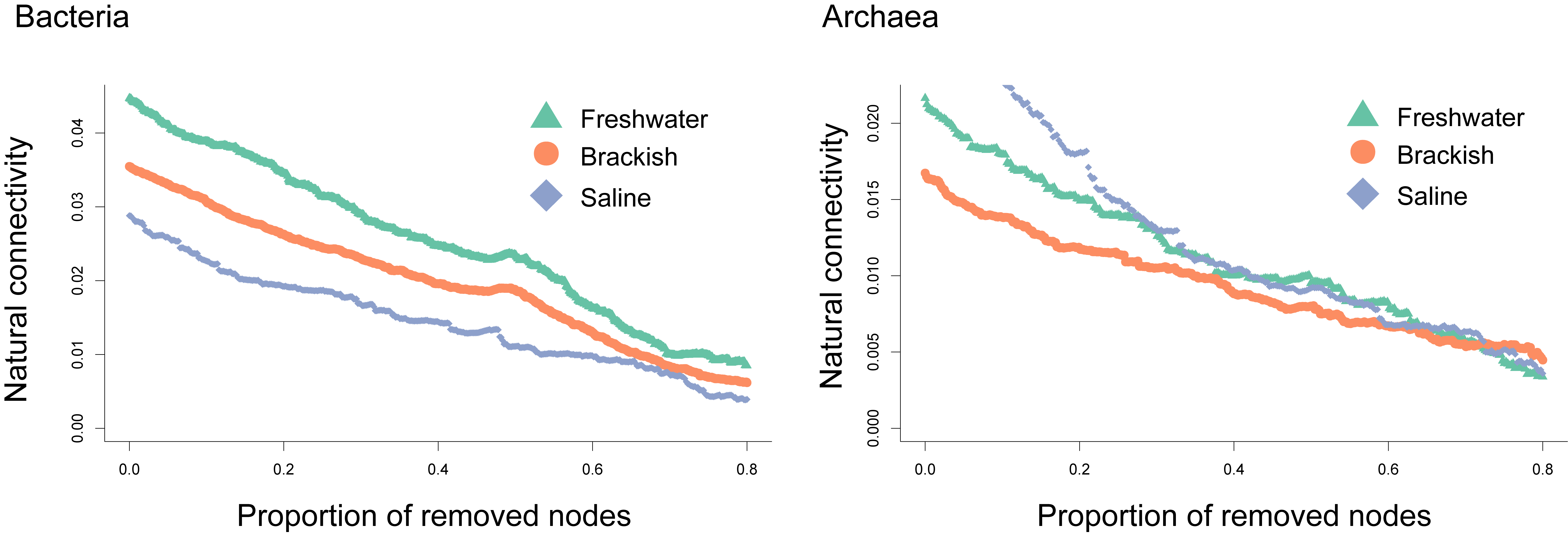
**

**Fig. S6.** Network stability across freshwater, brackish, and saline wetlands. Network fragility was calculated by natural connectivity after removing certain nodes, on behalf of network stability.


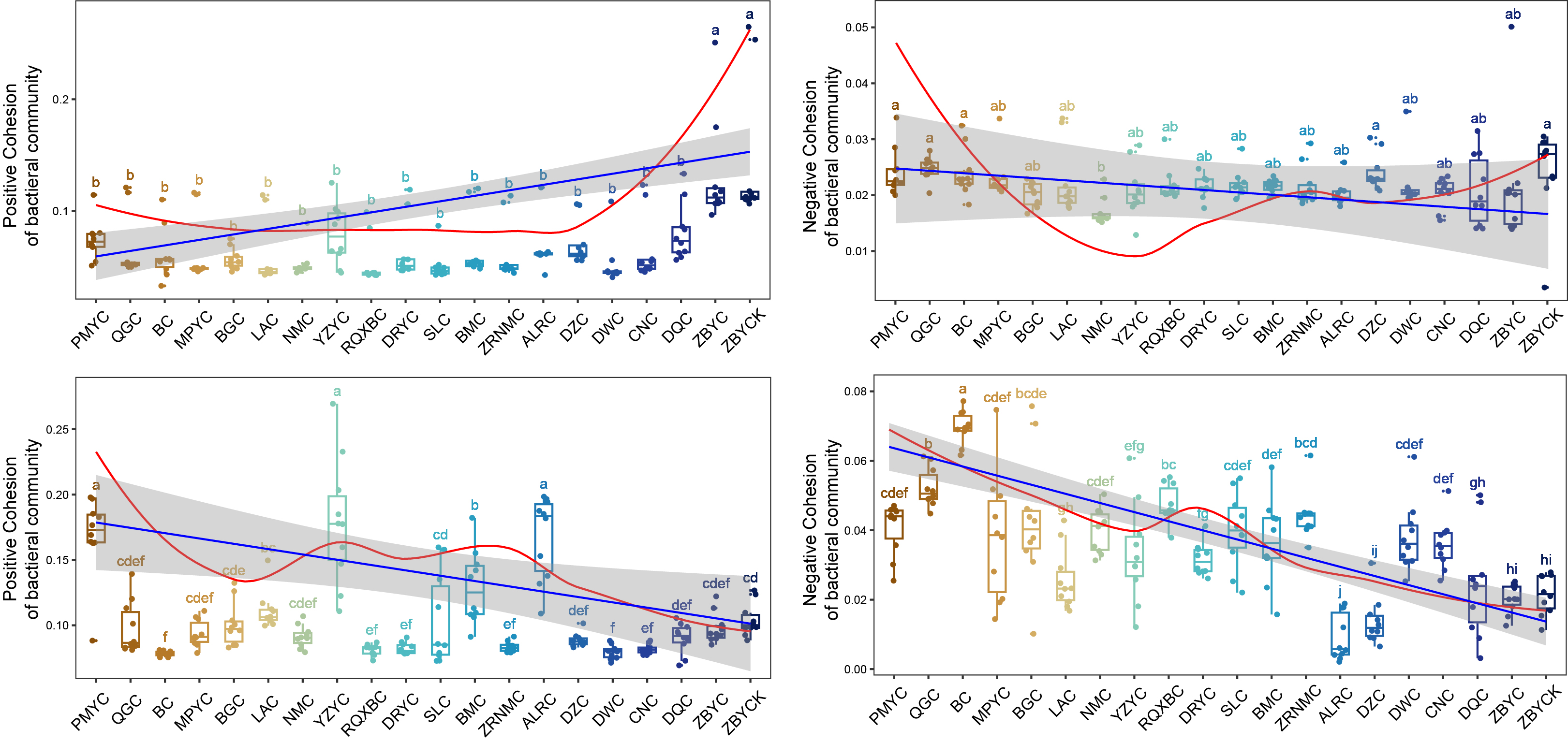


**Fig. S7.** Positive and negative cohesion for bacterial and archaeal communities across each alpine wetland. The results of Least Significant Difference (LSD) test were shown the among-group comparison in diagrams, with different letters denoting statistical significance. Statistical analysis for the relationship among site-level cohesion was performed using ordinary least squares linear regression (blue line) and loess non-linear regression (red line).


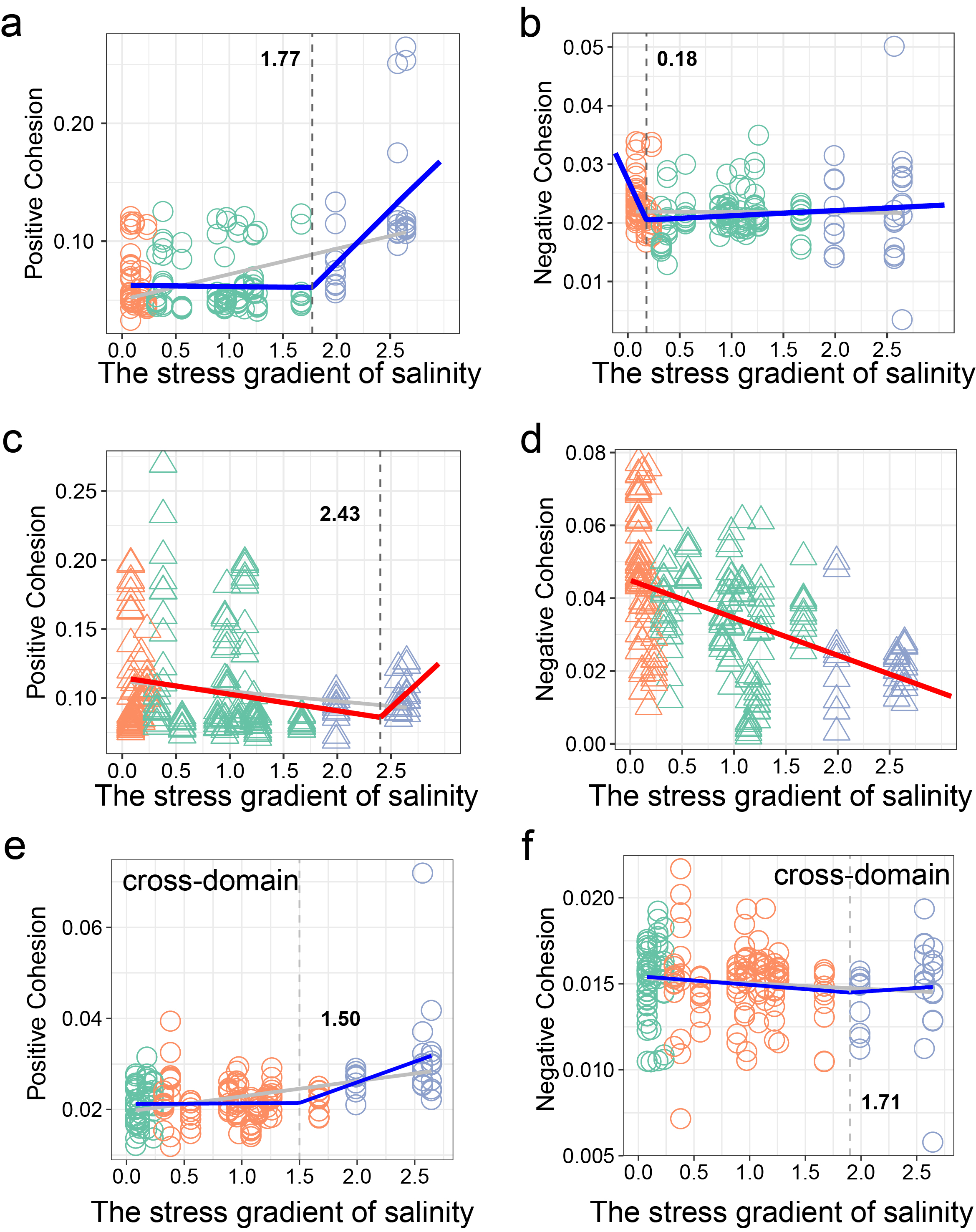


**Fig. S8. The non-linear relationships between salinity and ecological associations (network cohesion) of bacteria and archaea in the alpine wetlands of the Tibetan Plateau.** Non-linear responses of bacterial **(a–b)** and archaeal **(c–d)** ecological networks (positive and negative cohesion) to the salinity stress. Blue and red lines represent the trend fitted by a generalized additive model (GAM) and the linear fits at both sides of each threshold, respectively. Inset numbers and the vertical dashed lines describe the salinity thresholds identified. Statistics of non-linear regressions were also attached in the diagrams, corresponding to the R^2^ value and *P* significance. **e–f,** The response of cross-domain cohesion was also calculated.

**
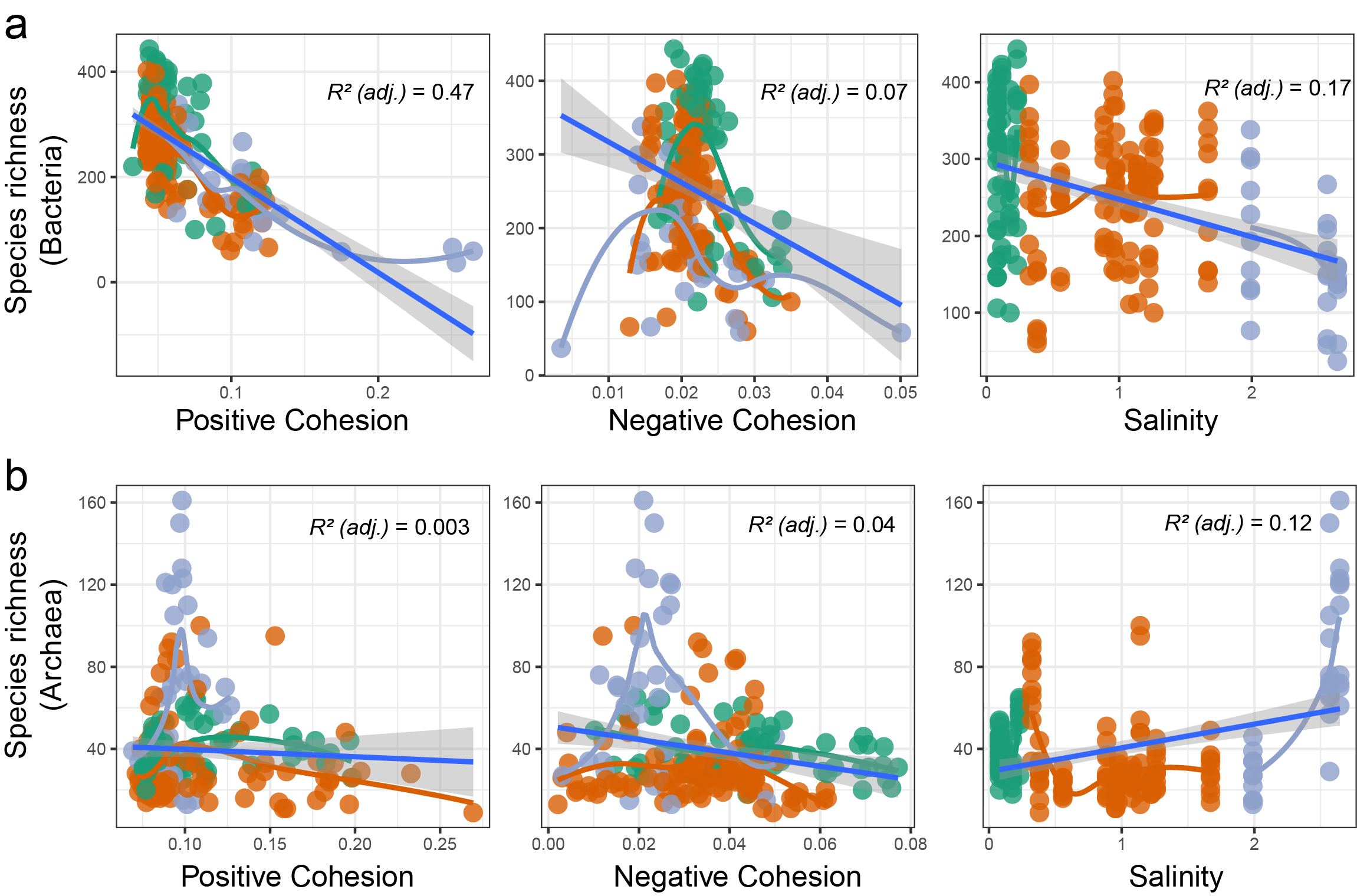
**

**Fig. S9.** Relationships between bacterial and archaeal diversity and network cohesions as well as salinity. Fitted linear relationships between driving factors (including positive cohesion, negative cohesion, salinity) and the richness of bacteria **(a)** and archaea **(b)** on the Tibetan Plateau. The relationships in freshwater (green), brackish (orange) and saline (blue) wetlands are also displayed by losses method. Statistical analysis for the relationship between driving factors and diversity was performed using ordinary least squares linear regressions. Adjusted R-squared results are also attached in the diagrams.


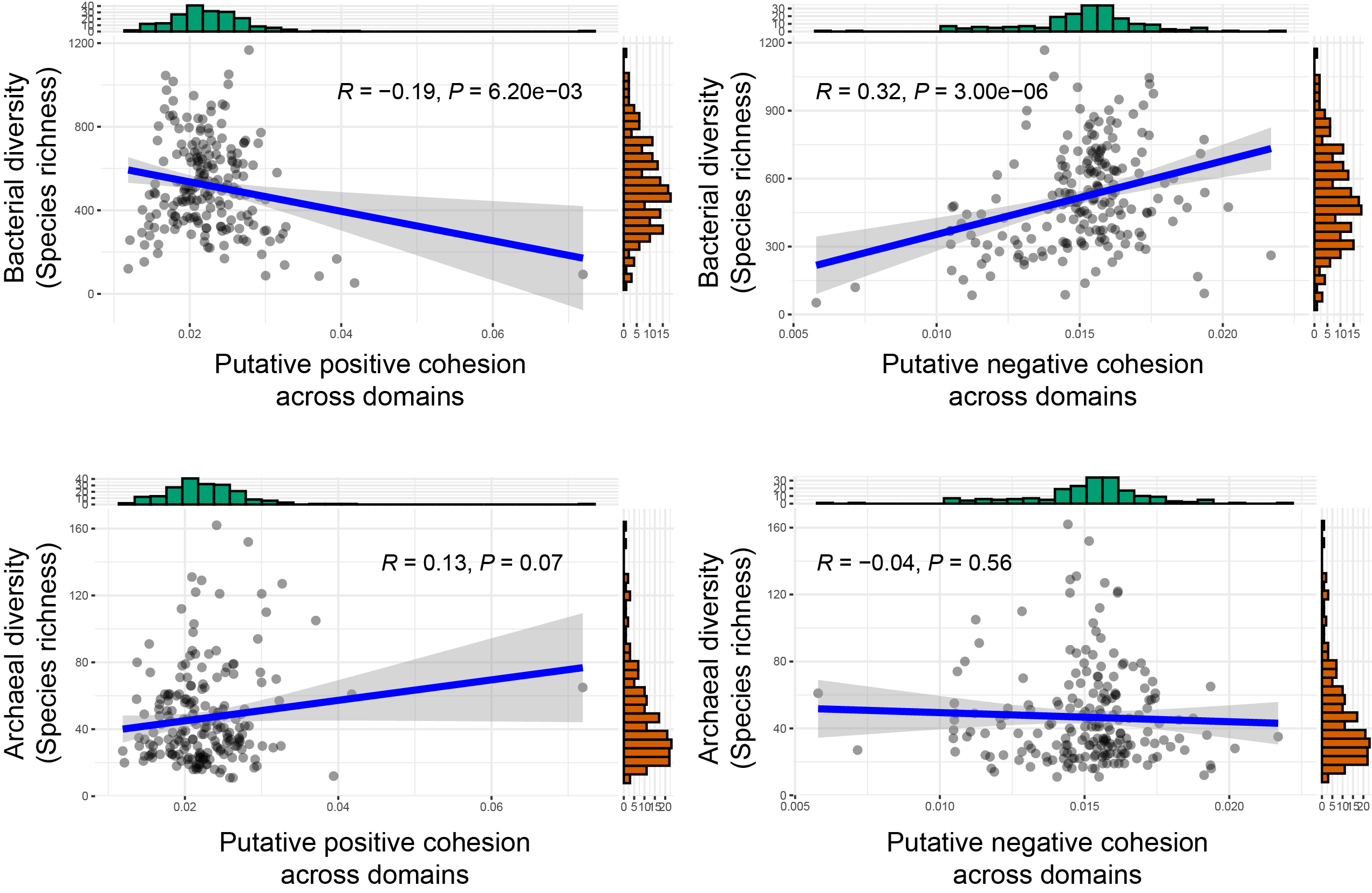


**Fig. S10.** The relationships between cross-domain cohesion and microbial diversity in the alpine wetlands on the Tibetan Plateau. The correlations were calculated according to positive and negative cohesion for bacteria and archaea, respectively. Statistics were also attached in the diagrams, corresponding to the R value and *P* significance.


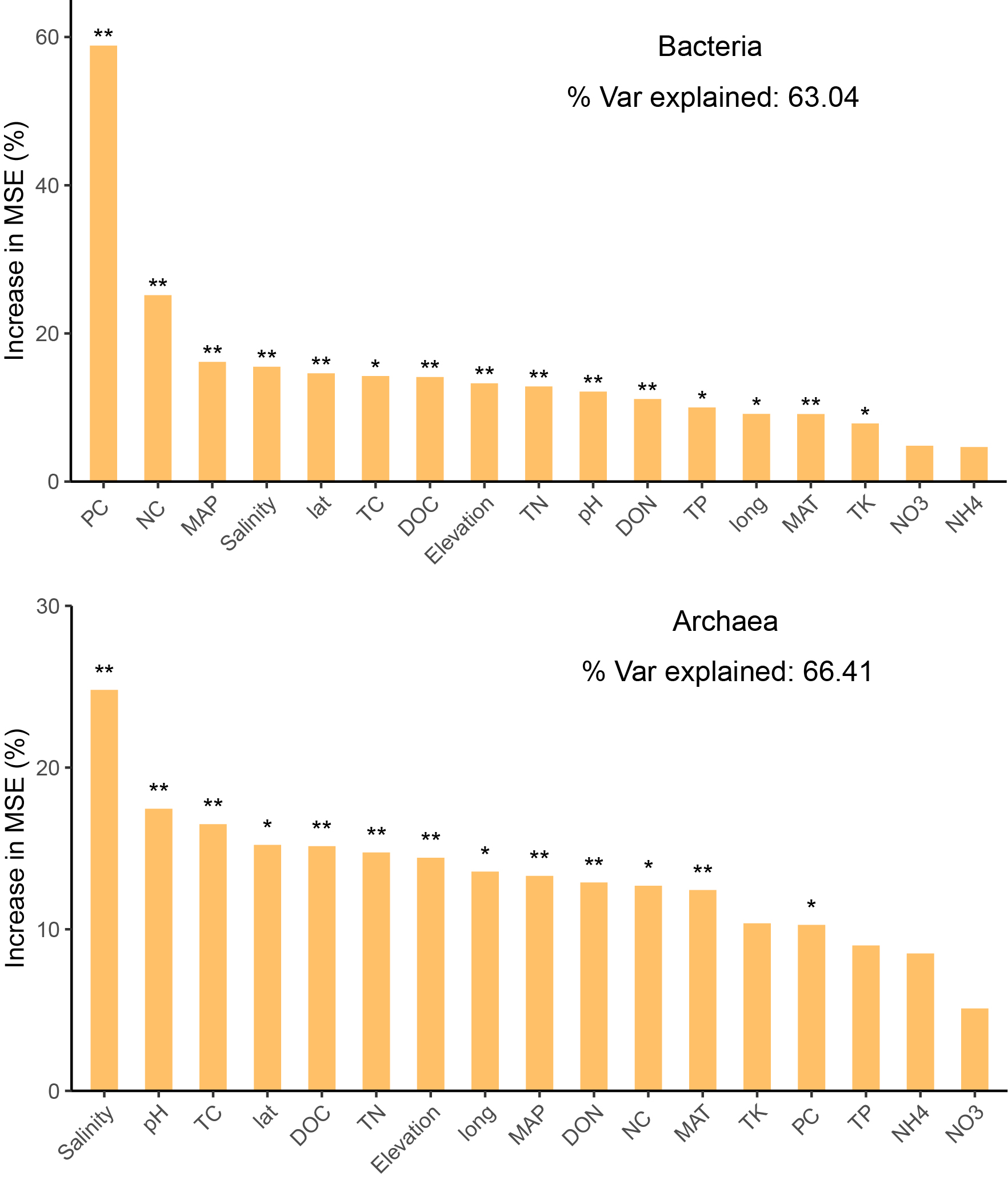


**Fig. S11.** Random forest (RF) means predictor importance (percentage of increase of mean square error) of soil, climate, space, salinity, and ecological association indices as drivers for species richness of bacteria and archaea in alpine wetlands, respectively. Percentage increases in the MSE (mean squared error) of variables were used to estimate the importance of these predictors, and higher MSE% values imply more important predictors. **P* < 0.05, ***P* < 0.01.


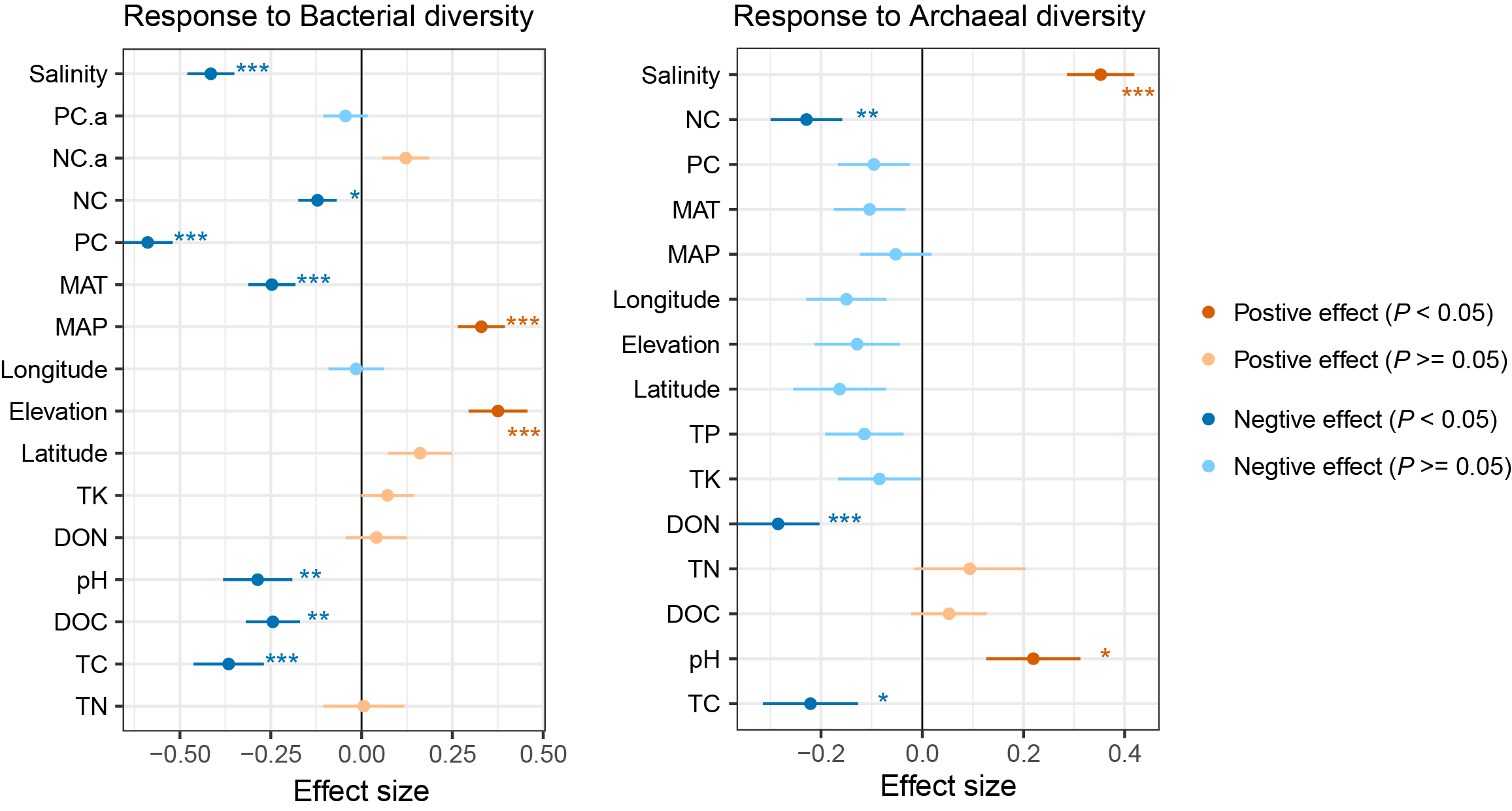


**Fig. S12.** The effect size of different explained variables responding to microbial diversity after controlling other factors in the structural equation model. Significance levels of each predictor are * *P* < 0.05, ** *P* < 0.01 and *** *P* < 0.001.
