# Supplementary material for: Contrasting patterns for bacteria and archaea in response to salt stress across alpine wetlands of the Tibetan Plateau
Source: Fundam Res. 2024 Mar 7;6(1):313–23. doi: 10.1016/j.fmre.2024.02.010 (PMC12869736; doi:10.1016/j.fmre.2024.02.010)
Supplement: Supplementary file 3 [file mmc3.docx]

**Supplementary Materials**

Tables S1 to S9

Figures. S1 to S12
